# Phylogeny and lipid profiles of snow-algae isolated from Norwegian red-snow microbiomes

**DOI:** 10.1093/femsec/fiad057

**Published:** 2023-05-24

**Authors:** Hirono Suzuki, Alexandre Détain, Youngjin Park, Kiron Viswanath, René H Wijffels, Nathalie Leborgne-Castel, Lenka Procházková, Chris J Hulatt

**Affiliations:** Faculty of Biosciences and Aquaculture, Nord University, 8049 Bodø, Norway; Faculty of Biosciences and Aquaculture, Nord University, 8049 Bodø, Norway; Faculty of Biosciences and Aquaculture, Nord University, 8049 Bodø, Norway; Faculty of Biosciences and Aquaculture, Nord University, 8049 Bodø, Norway; Faculty of Biosciences and Aquaculture, Nord University, 8049 Bodø, Norway; Bioprocess Engineering, AlgaePARC, Wageningen University, PO Box 16 Wageningen, 6700, AA, The Netherlands; Agroécologie, Institut Agro Dijon, CNRS, INRAE, Univ. Bourgogne Franche-Comté, F-21000 Dijon, France; Department of Ecology, Faculty of Science, Charles University, Viničná 7, 12844 Prague, Czech Republic; Centre for Phycology, Institute of Botany of the Czech Academy of Sciences, Dukelská 135, 37982 Třeboň, Czech Republic; Faculty of Biosciences and Aquaculture, Nord University, 8049 Bodø, Norway

**Keywords:** 18S rRNA, fatty acids, imaging flow cytometry, ITS2 rRNA, microalgae, phylogeny

## Abstract

Snow algae blooms often form green or red coloured patches in melting alpine and polar snowfields worldwide, yet little is known about their biology, biogeography, and species diversity. We investigated eight isolates collected from red snow in northern Norway, using a combination of morphology, 18S rRNA gene and internal transcribed spacer 2 (ITS2) genetic markers. Phylogenetic and ITS2 rRNA secondary structure analyses assigned six isolates to the species *Raphidonema nivale, Deuterostichococcus epilithicus, Chloromonas reticulata*, and *Xanthonema bristolianum*. Two novel isolates belonging to the family Stichococcaceae (ARK-S05-19) and the genus *Chloromonas* (ARK-S08-19) were identified as potentially new species. In laboratory cultivation, differences in the growth rate and fatty acid profiles were observed between the strains. Chlorophyta were characterized by abundant C18:3*n*-3 fatty-acids with increases in C18:1*n*-9 in the stationary phase, whilst *Xanthonema* (Ochrophyta) was characterized by a large proportion of C20:5*n*-3, with increases in C16:1*n*-7 in the stationary phase. In a further experiment, lipid droplet formation was studied in *C. reticulata* at the single-cell level using imaging flow cytometry. Our study establishes new cultures of snow algae, reveals novel data on their biodiversity and biogeography, and provides an initial characterization of physiological traits that shape natural communities and their ecophysiological properties.

## Teaser

Norwegian red snow microbiomes host phylogenetically diverse culturable microalgae that exhibit different growth strategies and accumulate lipids under physiological stress.

## Introduction

Microalgae inhabiting terrestrial snow environments are key primary producers in extreme ecosystems and are important for their biodiversity as well as interactions with climate warming (Gray et al. 2020, Krug et al. 2020). Snow algae blooms during spring and summer may form green, orange, or red coloured patches covering small (cm^2^) to large (∼km^2^) areas that can be observed in melting polar and alpine snowfields worldwide (Hoham and Remias 2020). Recently, snow algae have attracted a lot of attention due to their contribution to reducing the albedo of snow and ice surfaces, consequently accelerating snowmelt and causing positive feedbacks with a warming climate (Lutz et al. 2016, Cook et al. 2020). Despite their general macroscopic appearance, studies have revealed a substantial amount of global species diversity, much of which has not been characterized.

Red-snow communities are typically dominated by the Chlorophycean algae *Chloromonas, Chlainomonas*, and the newly described genus *Sanguina* (Procházková et al. 2019a). Amongst these communities, *Raphidonema* (Trebouxiophyceae) are also commonly observed (Lutz et al. 2016, Segawa et al. 2018, Engstrom et al. 2020). To study the biodiversity and biogeography of snow algae, metabarcoding approaches using common molecular markers have been applied. Lutz et al. (2016) used partial 18S ribosomal RNA (rRNA) gene-based analysis of samples from different regions of the Arctic and their data suggested that many groups of snow algae are cosmopolitan. Studies based on faster-evolving internal transcribed spacer 2 (ITS2) rDNA barcodes have also shown that polar red snow communities are typically dominated by a few cosmopolitan species, but also identified additional diverse species that are endemic to particular regions (Segawa et al. 2018). Although amplicon sequencing gives us insight into community structures, accurately distinguishing between closely related species or genera and evaluating biogeographical patterns of snow algae dispersal remain challenging, primarily due to lack of sample site coverage and limited phenotype/genotype reference data for high-throughput sequence analysis (Lutz et al. 2019). Alternatively, isolation and cultivation techniques allow us to characterize cell morphological and physiological properties together with sequence identity at high resolution. In the past, identification of algal species was often limited to morphological observation, but the combined use of molecular markers (18S, ITS2, and *rbc*L) as well as light and electron microscopy have greatly improved our understanding of cryospheric microflora by re-examining isolates and describing novel lineages and species (Hoham and Remias 2020). However, only a fraction of snow algae species, which are often in cyst form in the field, have been successfully cultured (Procházková et al. 2018a).

A few studies have described natural snow algae communities in mainland Norway (Kol 1963, Procházková et al. 2019a, Tucker and Brown 2022) and neighbouring Sweden (Kol 1974, Lutz et al. 2016). Kol (1963) observed snow algae communities at Finse (southwest Norway), where she found cells representing *Sanguina* (previously assigned to *Chlamydomonas nivalis* Wille), and *Raphidonema nivale* Lagerh. Procházková et al., (2019a) and Tucker and Brown (2022) recently characterized the dispersal of the *Sanguina* population worldwide, including environmental samples from southern Norway. In total, though, very little is known about snow algae biodiversity in this region, and no cultures are established from continental Norway.

Snow algae have diverse adaptations to the harsh, compound stress conditions found in melting snowfields (Leya 2013). In nature, they are often exposed to very high (inhibitory) levels of photosynthetically active radiation (PAR) coupled with elevated UV light levels, low nutrient concentrations, freeze-thaw cycles, and very low temperatures (Řezanka et al. 2008). Lipids are key molecules that perform structural, metabolic, and signalling functions in cell environmental adaptation, provide taxonomic markers, and distinguish the physiological status of communities in the field (Goold et al. 2015, Lutz et al. 2015, Davey et al. 2019, Lupette and Marechal 2020). The stress conditions prevalent in snow habitats are known to trigger neutral lipid and secondary carotenoid accumulation in cytoplasmic lipid droplets (LDs) in many algal species, including the characteristic red-coloured encysted snow-algae cells observed in nature (Leya et al. 2009, Procházková et al. 2019a). LDs in general are compartments that serve as a transient reserve of acyl-chains, and carotenoid-pigmented LDs may additionally have photoprotective roles by absorbing excessive light and scavenging/quenching reactive oxygen species (Britton et al. 2008, Li-Beisson et al. 2015). Assessment of snow algae lipids and their constituent fatty acids has received only limited research, yet lipids connect taxonomic variation with adaptive ecophysiological processes and more widely to the metabolic characteristics of whole snow algae communities.

In 2019, we obtained samples of red snow from melting summer snowfields near to Svartisen glacier, adjacent to the Arctic Circle in northern Norway, a region in which there are no earlier reports of snow-algae blooms. Our aim in this work was to isolate culturable eukaryotic microalgae from these field samples, to characterize the species present, and to establish them as laboratory cultures. Our primary questions were: (i) are culturable species found in this region globally dispersed cosmopolitan strains, or are they endemic to this region? (ii) how do lipid profiles and intrinsic growth rates, features that determine the construction and metabolic properties of snow algae communities, vary between isolates in diverse conditions?

## Materials and methods

### Sampling and algal strain isolation

**Figure 1.**
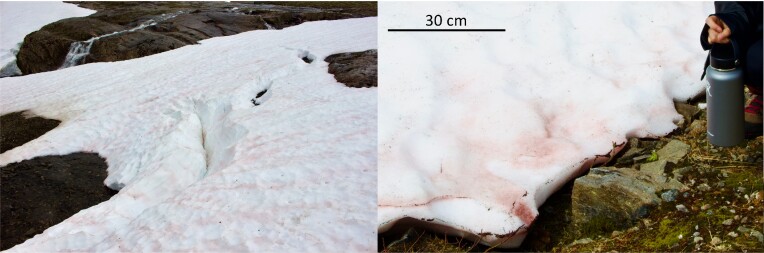
Sampling sites of macroscopically visible red-pigmented snow algae communities, Northern Norway. A 30 cm scale bar is shown (right).

Red-coloured snow was collected ∼1 km from Svartisen glacier in Northern Norway on June 23rd 2019 at an elevation of ∼540 m (66°45’03.9 N 14°05’55.0E). The sampling site is shown in Fig. 1. We have observed snow algae blooms on annual snowpack in this area each summer from 2019–2022, which appear in smaller patches (meters scale) dispersed around a wide area (several km^2^). Samples were collected in sterile plastic bags and transported to the laboratory within 4 h. Snow samples were thawed gently in a refrigerator at 4°C. Using a microscope, we observed a large proportion of red cells, interspersed with smaller numbers of diverse cell types (Fig. S1). To ensure we could obtain sufficient viable cells two isolation methods were used; (i) enrichment cultures followed by plating on agar and (ii) direct plating on agar. Enrichment cultures were prepared by filtering 500 ml of the thawed liquid samples onto ∼1.0 μm pore size 47 mm glass fiber filters (VWR, Norway). Filters were then added to a 500 ml Erlenmeyer flask containing autoclaved Bold's Basal Medium (BBM, Bischoff 1963, pH 6.5) and incubated at 6°C and 50 μmol·m^−2^·s^−1^ photons PAR in a plant growth chamber. After visible (green) cultures were established, culture broth from the flask was streaked onto 1% agar plates. When colonies appeared, each was transferred onto new plates. This procedure was repeated several times to obtain unialgal cultures. In parallel, thawed snow samples were spread directly on agar plates and colonies were selected as above. All isolates were then compared using microscopy (Olympus BX-43 microscope, Olympus Europa GmbH, Hamburg, Germany) to reduce redundancy and retain apparently unique strains based on cell size, morphology, the presence of cilia, chloroplast structure, and whether cells formed chains. Brightfield images were captured using × 1000 magnification and chlorophyll autofluorescence was imaged by excitation with blue LED light and an Olympus DP28 camera.

We selected eight isolates (named ARK-S*nn*-19, where *n* is a number) to study based on their morphological uniqueness. Cultivation for DNA extraction was performed in 250 ml Erlenmeyer flasks with BBM at 6°C at 20–40 μmol·m^−2^·s^−1^ photons PAR. Strains were also deposited at the Culture Collection of Cryophilic Algae, at the Fraunhofer Institute IZI-BB in Potsdam, Germany (http://cccryo.fraunhofer.de/web/strains/) (Table 1).

**Table 1.** Eight newly established algal isolates from snow assigned to species level, including NCBI GenBank accession numbers for the 18S rRNA gene and 5.8S rDNA partial + ITS2 rDNA + 28S rDNA partial sequences. Asterisk indicates the potentially new species. The isolates are deposited as corresponding ID in the Culture collection of Cryophilic Algae (CCCryo) in Germany.

Isolate number	Species designation		GenBank accession number (sequence length, bp)	
		CCCryo strain ID	18S rRNA gene	5.8S rDNA partial + ITS2 rDNA + 26S rDNA partial
ARK-S01-19	*Raphidonema nivale*	558–22	OM729978 (2173)	OM729984 (380)
ARK-S02-19	*Raphidonema nivale*	559–22	OM729979 (1649)	OM729985 (380)
ARK-S05-19	Stichococcaceae sp. ARK-S05-19 *	560–22	–	OM729986 (428)
ARK-S10-19	*Deuterostichococcus epilithicus*	561–22	–	OM729988 (395)
ARK-S08-19	*Chloromonas* sp. ARK-S08-19 *	562–22	OM729980 (1687)	OM729987 (437)
ARK-S11-19	*Chloromonas reticulata*	–	OM729981 (1603)	OM729989 (404)
ARK-S12-19	*Chloromonas reticulata*	563–22	OM729982 (1631)	OM729990 (404)
ARK-S13-19	*Xanthonema bristolianum*	564–22	OM729983 (1708)	–

### DNA extraction, polymerase chain reaction (PCR), and sequencing

For molecular identification of each strain, we attempted to sequence both the 18S and ITS2 rDNA. The 18S rRNA gene analysis, including extraction and amplification by PCR was performed by Roscoff Culture Collection (Roscoff, France). Genomic DNA was extracted with the Nucleospin Tissue Kit (Macherey-Nagel, Düren, Germany). The quality and quantity of DNA were measured by NanoDrop ND-1000 (Thermo Scientific, MI, USA). PCRs were conducted using 1 μL of DNA extract and 29 μL of a mixture of HotStarTaq Plus Master Mix Kit (Qiagen, Hilden, Germany), Coral Load (from the kit), primers, and H_2_O (15/3/0.5/0.5/10, v/v/v/v/v). The small subunit (18S) rRNA gene was amplified using universal eukaryotic primers (Euk-A, forward): 5′-AACCTGGTTGATCCTGCCAGT-3′, (EukBr, reverse): 5′-TGATCCTTCTGCAGGTTCACCTAC -3′ (Medlin et al. 1988), (NSF573, forward): 5′-CGCGGTAATTCCAGCTCCA-3′ (Hendriks et al. 1989), and (S69R, reverse): 5′-CCGTCADTTCCTTTRAGDTT-3′ (Probert et al. 2014). Initial denaturation at 95 °C for 5 min was followed by 35 cycles of denaturation at 95°C for 30 s, annealing at 52°C for 30 s, extension at 72°C for 1.5 min, and final extension at 72 °C for 15 min. PCR products were purified using ExoSAP-IT^TM^ PCR Product Cleanup Reagent (Thermo Scientific, Waltham, United States) and sequenced by Macrogen (Amsterdam, Netherlands).

For ITS2 rDNA sequencing, DNA was extracted from wet cell pellets using an E.Z.N.A.® HP Plant DNA Kit (Omega Bio-tek, Georgia, USA). Bead milling (4000 r/m × 5 min, Precellys 24, Bertin Technologies, Montigny le Bretonneux, France) and 0.1 mm glass beads were used to assist cell lysis. The DNA amount and quality were determined by Nanodrop One (Thermo Scientific, MI, USA). Amplicon library preparation and Illumina sequencing were performed by BGI (Guangdong, China) using 300 bp paired end reads on a HiSeq2500 instrument. The forward primer ITS3: 5′-GCATCGATGAAGAACGCAGC-3′ and reverse primer ITS4: 5′-TCCTCCGCTTATTGATATGC-3′ (White et al. 1990) were used for fragment amplification. The overlapping read pairs from each fragment were fused with FLASH (Magoč and Salzberg 2011), yielding the 5.8 rDNA partial + ITS2 + 28S rDNA partial sequence from seven out of the eight isolates (Table 1). The sequences were clustered with ‘cd-hit-est’ at setting ‘-c 1.00 -sc 1’ to quantify the unique sequences and eliminate the possibility of other eukaryotic contaminants (Li and Godzik 2006). The 5.8 rDNA partial + ITS2 + 28S rDNA partial, and 18S rRNA gene sequences were deposited in GenBank (Table 1).

### Phylogenetic analysis

Phylogenetic trees of *Raphidonema*-like strains were constructed based on the sequence alignment of the 18S rRNA gene and alignment of ITS2 rRNA secondary structure, respectively, whilst that of *Stichococcus*-like strains was constructed based only on alignment of ITS2 rRNA sequence structure. Two phylogenetic trees of *Chloromonas*-like strains were constructed based on the sequence alignment of 18S rRNA gene and ITS2 rDNA, whilst that of *Xanthonema*-like strains was constructed based only on 18S rRNA gene sequences. The 18S and ITS2 rDNA sequences of related strains were obtained from NCBI GenBank using BLASTn queries (http://blast.ncbi.nlm.nih.gov, Altschul et al. 1990). The sequences were aligned with MUSCLE (multiple sequence alignment by log-expectation) in MEGAX 10.1.8 (Kumar et al. 2018), then visualized and trimmed to obtain the same lengths for all sequences. The 18S rRNA gene alignment of *Raphidonema, Chloromonas*, and *Xanthonema* contained 45 (1661 bp), 52 (1553 bp) and 23 sequences (1730 bp), respectively. The ITS2 rRNA matrix (secondary structure alignment) of *Raphidonema* and *Stichococcus*-like species consisted of 28 (354 bp) and 41 sequences (396 bp), respectively. The ITS2 rDNA alignment of the *Chloromonas* tree contained 16 sequences (321 bp). Phylogenetic trees were constructed using maximum likelihood (ML) with the best model predicted based on BIC in MEGAX v10.1.8. Outgroups and the selected optimal model are specified in each figure or the legend of the corresponding phylogenetic tree. The bootstrapping values (1000 replicates) and Bayesian posterior probabilities were calculated by MEGAX 10.1.8 and MrBayes 3.2.6 (Ronquist and Huelsenbeck 2003), respectively.

### ITS2 rRNA secondary structure analysis

The secondary structure of the ITS2 rRNA was analyzed to examine species boundaries between *Raphidonema* strains, between S*tichococcus*-like strains, and between ARK-S08-19 and *Chloromonas augustae* SAG 5.73. The ITS2, 5.8S, and large subunit (LSU) stem region of rDNA was annotated using the ITS2 database (Keller et al. 2009). The ITS2 secondary structures were computed using a minimum free energy prediction model with the Mfold server (Zuker 2003). Predicted structures were visualized and only sequence-structures containing the specific features of ITS2 rRNA such as four helices with UGG and GGU motifs and U-U mismatch (Coleman 2007, Caisová et al. 2013) were selected for further analysis. The selected sequence-structure was aligned using ClustalW, and the compensatory base pair changes (CBCs) were computed and visualized in 4SALE 1.7 (Wolf et al. 2014). The aligned sequence-structure data were exported to MEGAX 10.1.8. The secondary structure was drawn by Visualization Applet for RNA (Varna) version 3.8 (Darty et al. 2009) or 4SALE 1.7, followed by editing with Inkscape 0.92 (http://www.inkscape.org/).

### Cultivation experiments

Three different cultivation experiments were performed to target growth rates, fatty acids and LD accumulation. Each of the experiments was conducted in a temperature-controlled plant growth incubator fitted with cool white fluorescent lights (Termaks AS, Norway). Cultivation vessels and nutrient medium were sterilized by autoclaving (121°C, 20 min).

### Cell density

The cell density was measured by the optical density (OD), which was calibrated against the dry weight (DW). The OD was measured at 540 nm, which avoided chlorophyll absorbance peaks. A 1 cm cuvette was used for OD measurement, with samples diluted below absorbance 1.0 for linear response. In Experiments 1 and 3, the DW was measured at the beginning and end of cultivation by filtering 3–10 ml of culture broth through pre-weighted ∼1.0 μm pore size 47 mm glass fiber filters (VWR, Oslo, Norway). Filters were dried at 100°C for 24 h, then re-weighed and the DW (g·L^−1^) was calculated. For each species, linear calibration was used to calculate the DW from the OD (Fig. S2).

### Experiment 1. Growth rates

The eight isolates were cultivated at 2°C and at 10°C in glass tubes (*n* = 3 at each temperature), containing 90 ml of 3N-BBM (BBM with 3-fold nitrate, Bischoff 1963, pH 6.8) and illuminated with 109 ±5 μmol·m^−2^·s^−1^ photons PAR. Each tube was sparged with air enriched with 1.0% CO_2_ at 62.5 ml·min^−1^, supplied by a gas mixing system (Photon Systems Instruments GMS 150, Drásov, Czech Republic). OD measurements were taken every 1–2 days and at the end of experiments cells were pelleted and stored at –40°C for further analysis.

The growth rate of each strain was established by fitting the cell density measurements to a logistic growth equation using the R package ‘*Growthcurver*’ (Sprouffske and Wagner 2016) in R software version 3.1.2 (Team, 2018).


(1) \begin{eqnarray*} {N_t} = \frac{K}{{1 + \left( {\left. {\frac{{K - {N_0}}}{{{N_0}}}} \right){e^{ - rt}}} \right.}}, \end{eqnarray*}

where, *N_t_* is the population size (cell density, g·L^−1^) at time *t* (days), *N_0_* is the initial cell density (g·L^−1^), *K* is the carrying capacity (maximum cell density, g·L^−1^) and *r* is the population growth rate (μ·d^−1^). Growth curves were fitted independently to each replicate cultivation, and the mean ± SD of *n* = 3 is presented for each of the eight isolates at each temperature.

### Experiment 2. Fatty-acid profiling

To determine the fatty-acid profile of each isolate under standardized non-stressed conditions, cells were cultured at 6°C under 50 μmol·m^−2^·s^−1^ photons PAR in 500 ml Erlenmeyer flasks (*n* = 4 each) containing 200 ml of BBM (pH 6.8). Cells were harvested by centrifugation (3,000 *g*, 3 min) when the OD reached absorbance 0.25, ensuring the cell density was well below the carrying capacity of the nutrient medium and each culture received equivalent light supply at the time of collection. Samples were stored at −40 °C.

### Experiment 3. LD accumulation in ARK-S12-19

Strain ARK-S12-19 was cultivated at 10°C in 27 mm diameter glass bubble tubes containing 380 ml of 3N-BBM (pH 6.8). The tubes were supplied with air containing 1.0% CO_2_ at 85 ml·min^−1^ and 145 ±8 μmol·m^−2^·s^−1^ photons PAR. The cells were first grown for 9 days, then the cultures were gently centrifuged (1920 *g*, 15 min) and resuspended in nitrate (N)-free media to initiate the start of the experiment (day 0), providing an accurate onset of N-starvation. The subsequent experimental cultivation period in N-free medium was 11 days, with biomass, imaging flow cytometry and lipid samples collected at day 0, 1, 2, 4, 7, and 11.

### Imaging flow cytometry

An ImageStream^®X^ Mk II Imaging Flow Cytometer (Luminex Corporation, Austin, TX, USA) was used to measure cell dimensions, LD accumulation and chlorophyll content in Experiment 3. Cell LDs were labelled with Bodipy^®^ 493/503 (Molecular Probes, Eugene, OR, USA) dissolved in 100% DMSO (v/v) at a final concentration of 0.075 μg mL^−1^, mixed and incubated for 15 mins. A 488 nm laser operated at 1.0 mW was used for excitation of the probe and chlorophylls. Detector wavelengths for the different channels were 505–560 nm (Bodipy^®^ label, Channel 2); 595-642 nm (brightfield image, Channel 4); 642-745 nm (chlorophyll autofluorescence, Channel 5). A total of up to 10000 particles were analyzed at a rate of ∼50 objects/second at magnification × 40. IDEAS v.6.1.823.0 software was used for downstream analysis, including gating to select the main population of single cells and exclude debris or doublets. Image masking was conducted to measure the length, width, area and fluorescence intensity of each individual cell. Cell dimensions and area were measured from the brightfield images (Channel 4) by applying an image mask, and the fluorescence intensity of Channel 2 and Channel 5 were determined from the same mask area (Fig. S13). The biovolume of each cell was calculated from the length/width dimensions of the image using the formula for a prolate spheroid shape (Hillebrand et al. 1999).


(2) \begin{eqnarray*} V = \frac{\pi }{6} \cdot {d^2} \cdot h, \end{eqnarray*}

where, *V* is the volume (μm^3^), *d* is diameter (μm), and *h* is height (μm) of the cell. Both the cell area and cell volume were tested as normalization values, by dividing Bodipy^®^ fluorescence by either the area or volume.

### Extraction, isolation of lipids, and methylation

For Experiment 1, we directly extracted and derivatized the fatty acids to fatty-acid methyl esters (FAMEs) from cells cultivated at 10°C as described by Radakovits et al. (2012). For Experiments 2 and 3, total lipids were extracted from freeze-dried cell pellets using chloroform and methanol as described by Breuer et al. (2012). For Experiment 3, neutral lipids and polar lipids were additionally separated by solid-phase extraction (Sep-Pak 1 g silica cartridges, 6 ml, Waters, MO, USA) followed by the separation of TAG (Triacylglycerol) from neutral lipids by high-performance thin-layer chromatography (HPTLC). Fatty acids in each lipid fraction were derivatized by adding 3 ml of 5% H_2_SO_4_ in methanol and incubation at 70°C for 3 h. After washing with NaCl-saturated water, FAMEs were recovered in hexane. For a full description of the experiments, see “Supplementary Material” section.

### FAME analysis

FAMEs were quantified by a Gas Chromatograph (GC) equipped with a Flame Ionization Detector (FID, SCION 436, Bruker, UK) and an Agilent CP-Wax 52CB column (Agilent Technologies, USA). Supelco 37 component standards (Sigma–Aldrich, MA, USA) were used for identification and quantification of the FAMEs, with additional standards for unsaturated C16 and C18 fatty acids. To identify unusual fatty acids, the FAMEs structures of ARK-S01-19 and ARK-S13-19 were verified by Helsinki University Lipidomics Unit (HiLIPID). The analyses were performed based on their electron-impact mass spectra recorded by GC-MS-QP2010 Ultra (Shimadzu Scientific Instruments, Kyoto, Japan), compared to the spectra of analytical standard mixtures of FAMEs (Supelco® Analytical Products, Merck) and published reference mass spectra (Christie 2021). The GC-mass spectrometer (GC-MS) was equipped with a Zebron ZB-wax capillary column (30 m, 0.25 mm ID and film thickness 0.25 μm; Phenomenex, Torrence CA, USA), which gave the FAMEs similar retention time patterns as the column used for the quantitative GC-FID analyses.

## Results

### Snow-algae identification: cell morphology and phylogeny

**Figure 2.**
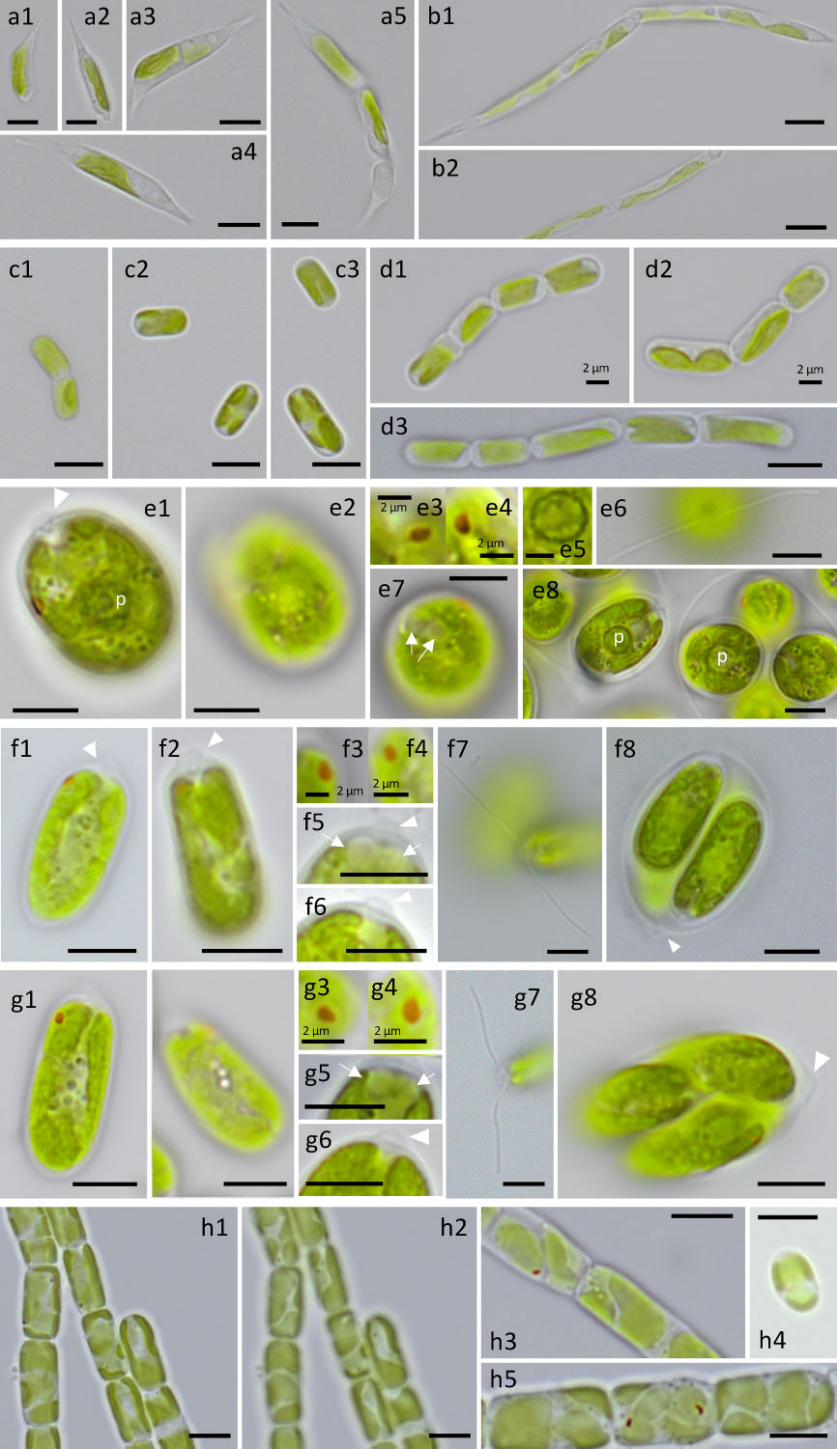
Microphotographs of eight algal isolates: *R. nivale* ARK-S01-19 (a1, a2, a3, a4, a5); *R. nivale* ARK-S02-19 (b1, b2); Stichococcaceae sp. ARK-S05-19 (c1, c2, c3); *D. epilithicus* ARK-S10-19 (d1, d2, d3); *Chloromonas* sp. ARK-S08-19 (e1, e2, e3, e4, e5, e6, e7, e8); *C. reticulata* ARK-S11-19 (f1, f2 f3, f4, f5, f6); *C. reticulata* ARK-S12-19 (g1, g2, g3, g4, g5, g6, g7, g8); *X. bristolianum* ARK-S13-19 (h1, h2, h3, h4, h5). Arrowheads indicate the papillae and arrows indicate contractile vacuoles. Scale bars are either 2 μm (with indication of the scale size near the bar) or 5 μm (no indication of the scale size). p = pyrenoid.

Morphological features (Fig. 2, Fig. S3) analyzed using light microscopy together with sequence data of molecular markers were used to identify eight snow algae isolates. We constructed different phylogenetic trees to place the genus *Raphidonema* (2 isolates), *Chloromonas* (3 isolates), *Xanthonema* (1 isolate), and the family Stichococcaceae (2 isolates) using the sequences presented in Table 1.

### *Raphidonema*-like species

The cells of ARK-S01-19 were filiform or elongated fusiform, straight 8.1–45.4 μm long and 2.0–3.9 μm wide with bent aristate or rotund apices (Fig. 2 a1–a5). Cells were usually unicellular, but sometimes remained united in short chains up to four cells, with ribbon-like chloroplasts (Fig. S3a). Filaments with branched acute ends were observed. ARK-S02-19 cells were filiform, straight 15.1–47.6 μm in length and 2.3–3.8 μm in width with acute apices or rotund apices (Fig. 2 b1and b2). The cells commonly formed unbranched filaments consisting of more than four and up to sixteen cells with plate-like chloroplasts (Fig. S3b).

**Figure 3.**
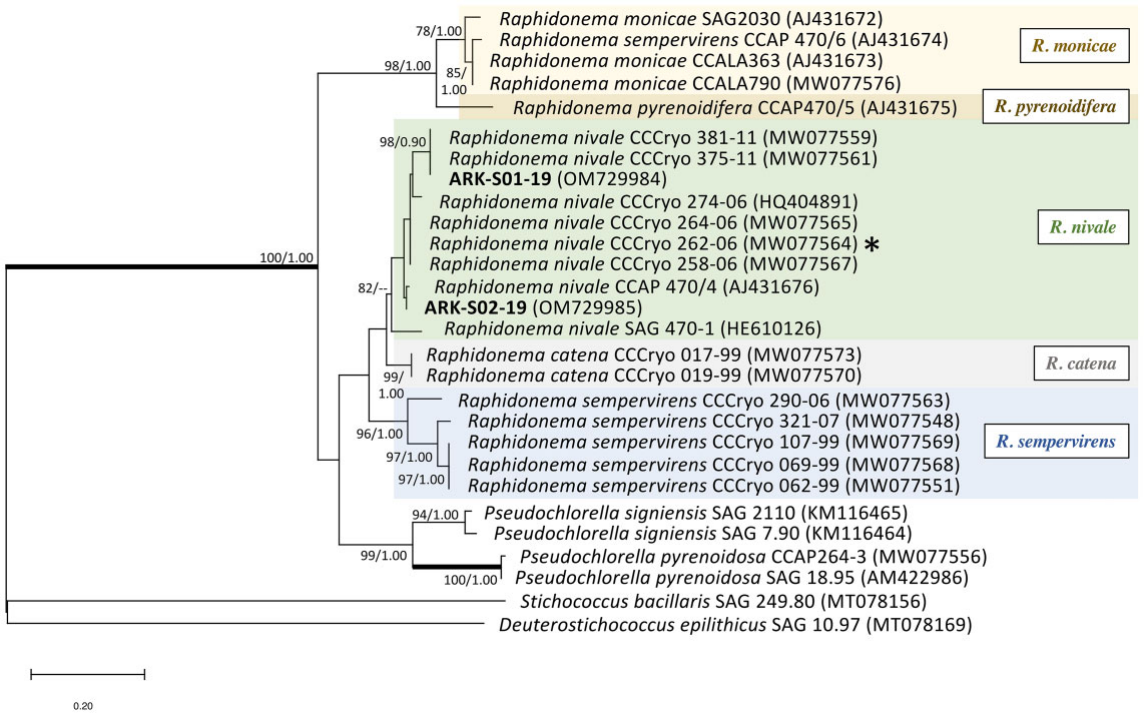
ITS2 rRNA sequence-structure ML tree of *Raphidonema* with the new isolates (sequence) shown in bold. An asterisk indicates the type strain of the species *R. nivale* and the species names are as proposed by Yakimovich et al. (2021). The best model was JTT+G+I+F calculated by MEGAX 10.1.8. Numbers next to branches indicate statistical support values [ML bootstraps (1000 replicates)/Bayesian posterior probabilities]. The bootstrap support values above 70% and Bayesian posterior probabilities above 0.90 are shown. Thick lines indicate branches with full statistical support (ML/BI:100/1.00). *Stichococcus bacillaris* SAG 249.80 (MT078156) and *D. epilithicus* SAG 10.97 (MT078169) serve as the outgroup.

The ITS2 rDNA sequence of ARK-S01-19 was identical to *R. nivale* (Lagerheim) CCCryo 381–11 and CCCryo375-11 isolated from a red snowfield (77°0’N 16°37’E) and on top of a steep snowfield on Svalbard (77°0’N 16°22’E), respectively. The 18S rRNA gene sequence of ARK-S02-19 was identical to *R. nivale* strain CCAP 470/4 isolated from a mountain at an altitude of 1295 m in Washington State (USA), with only 1 bp difference in ITS2 rDNA. We constructed a ML tree based on 18S rRNA gene sequences, which revealed that each isolate formed a different subclade together with other *Raphidonema* within a clade with full statistical support (ML/BL:100/1.00), being a sister to the strains identified as *Pseudochlorella* (Fig. S4). ITS2 rRNA sequence-structure analysis was further used for species-level resolution, where ML analysis showed that our two isolates fell into a clade together with other *R. nivale* (ML:82) (Fig. 3). The worldwide distribution of *Raphidonema* strains in the phylogenetic tree (Fig. 3) is mapped in Figure S5, indicating the geographic location of the closest relatives to the new isolates and highlighting the known distribution of *R. nivale* in maritime Antarctica (James Ross), Western USA, Svalbard, continental Norway, and Switzerland.

### *Stichococcus*-like species

The two *Stichococcus*-like isolates ARK-S05-19 and ARK-S10-19 are both cylindrical cells with rounded ends (Fig. 2C and D). The cells of ARK-S05-19 are 4.1–8.9 μm in length and 2.3–3.6 μm in width, and the length to width ratio was around 2.2 (Fig. 2 c1–c3). The cells were usually unicellular, but sometimes occurred as unbranched two-cell filaments with plate-like chloroplasts (Fig. S1c). In contrast, ARK-S10-19 cells were 5.2–14.8 μm long and 2.2–3.2 μm wide, resulting in the length to width ratio of 3.0, and usually unicellular but occasionally appearing as short unbranched filaments of 2 to 8 cells with plate-like chloroplasts (Fig. 2 d1–d3 and Fig. S3d).

**Figure 4.**
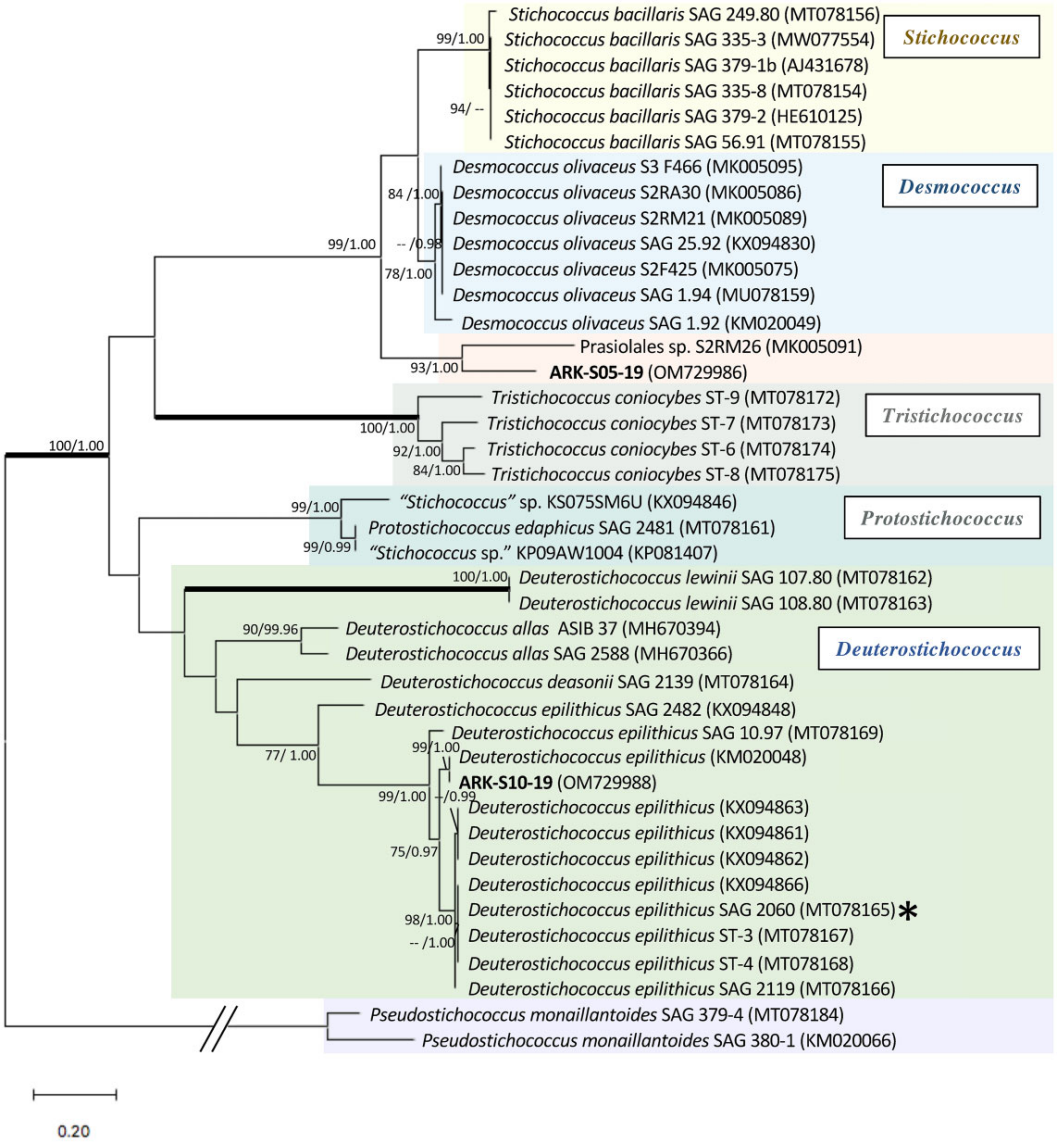
ITS2 rRNA sequence and secondary structure-based ML phylogeny of *Stichococcus*-like species with the new isolates (sequences) shown in bold. The best model was WAG+G+F, calculated by MEGAX 10.1.8. The asterisk shows the type strain *Deusterostichococcus epilithicus* according to Pröschold and Darienko (2020). Numbers next to branches indicate statistical support values [ML bootstraps (1000 replicates)/Bayesian posterior probabilities]. The bootstrap support values above 70% and Bayesian posterior probabilities above 0.9 are shown. Thick lines indicate the branches with full statistical support (ML/BI:100/1.00). *Pseudostichococcus monaillantoides* SAG 379–4 (MT078184) and *P. monaillantoides* SAG 380–1 (KM020066) serve as the outgroup.

**Figure 5.**
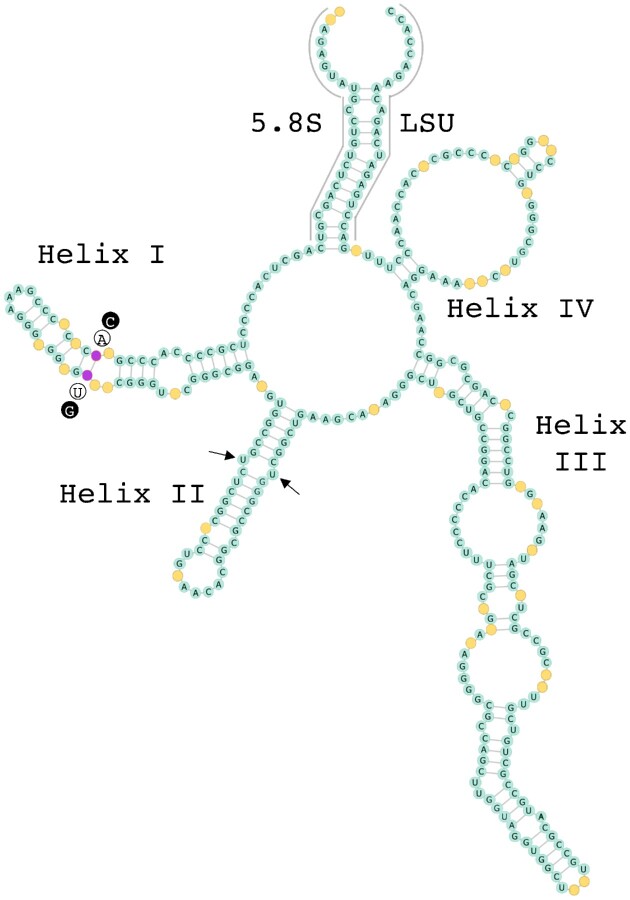
The consensus structure of ITS2 rRNA sequence secondary structure between ARK-S05-19 (OM729986) and Prasiolales sp. S2RM26 (MK005091). Partial 5.8S and partial LSU stem regions of rRNA are included. One compensatory base change (CBC) between these two species within helix I is indicated in pink. Nucleotides highlighted in circles outside the yellow mark indicates a CBC between ARK-S05-19 (white circles) vs Prasiolales sp. S2RM26 (black circles). The conserved regions are indicated in blue with nucleotides (A, U, G, or C) and non-conserved nucleotides are shown in yellow. The ITS2 secondary structure and sequences were synchronously aligned and visualized using 4SALE.

The 18S rRNA gene sequences of *Stichococcus*-like species were unfortunately unobtainable due to unsuccessful amplification by PCR (Table 1). However, the ITS2 rRNA sequence-structure based phylogenetic tree showed that ARK-S10-19 belonged to a *Deuterostichococcus* clade (Pröschold and Darienko 2020). Furthermore, its 5.8S rDNA partial + ITS2 + 28S rDNA partial sequences were identical to that of *D. epilithicus* FG2/4.2 (KM020048), for which the isolation/sample location or habitat are unknown. *Deuterostichococcus epilithicus* have been found worldwide, many of which are isolated from soil or subaerial habitats (Fig. S6). In contrast, the closest ITS2 rDNA of ARK-S05-19 in the NCBI database was the unclassified isolate Prasiolales sp. S2RM26, originating from the surface of snowfall samples in Sweden (Tesson and Šantl-Temkiv 2018), revealing a difference of 49 nucleotides out of 318 (85% identity). ARK-S05-19 and the unclassified isolate Prasiolales sp. S2RM26 together formed a well-supported (93%) independent clade distant from the sister lineages *Desmococcus olivaceus* and *S. bacillaris* (Fig. 4). Nevertheless, one CBC difference was detected in helix I between ARK-S05-19 and Prasiolales sp. S2RM26 (Fig. 5). Several strains of *S. bacillaris* and five strains of *D. olivaceus* also provided the highest BLAST matches for the ITS2 rDNA sequence of ARK-S05-19, but their rRNA secondary structures differed by three and four CBCs, respectively (Table S1, Table S2).

### *Chloromonas*-like species

**Figure 6.**
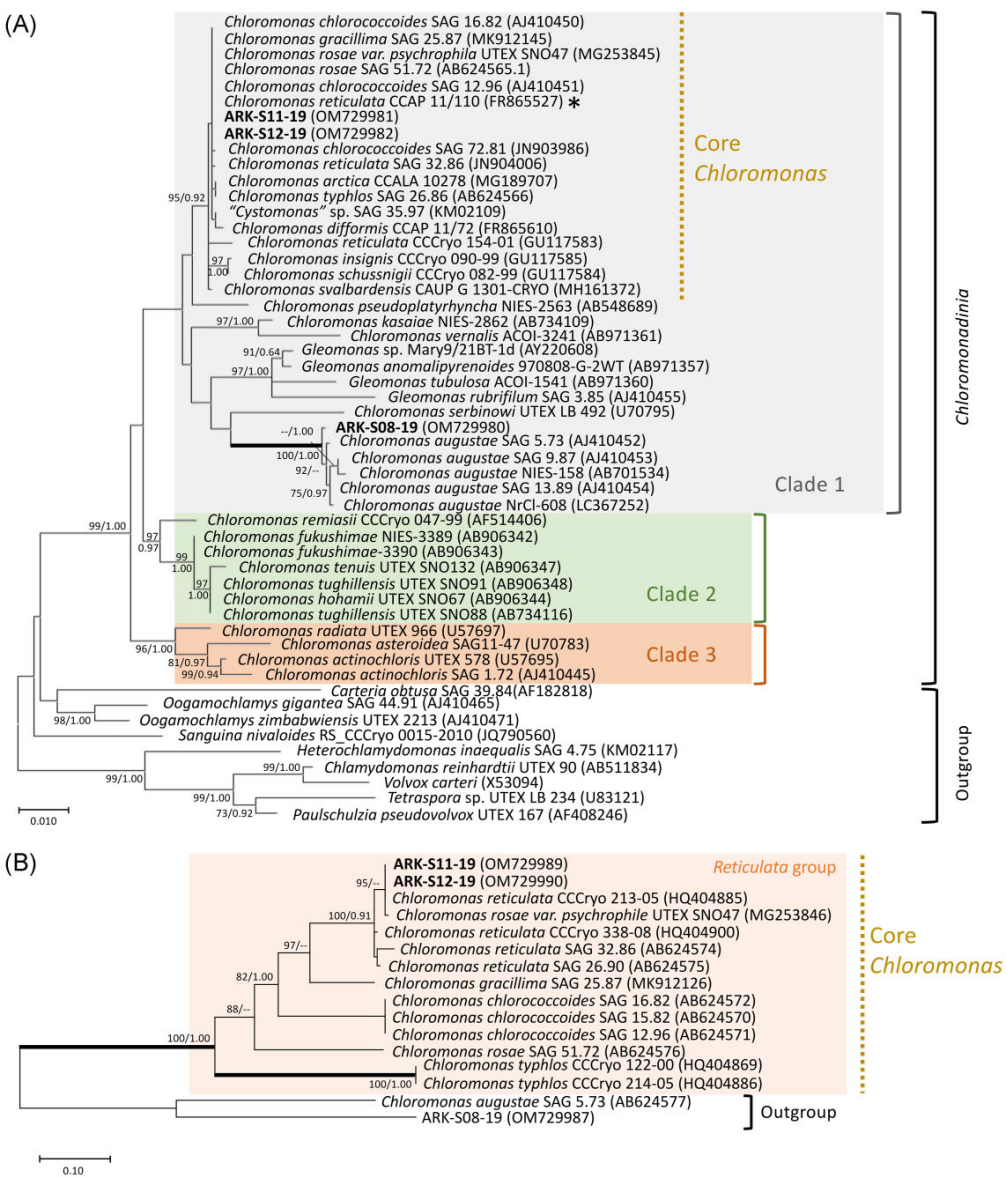
**(A)** 18S rRNA gene-based ML phylogenetic tree of *Chloromonas*. The asterisk shows the authentic strain of *C. reticulata*, the type species of the genus *Chloromonas*. Our new isolates (sequence) are shown in bold. *Chloromonas* clade 1,2 and 3 are delimited according to Hoham et al. (2002). **(B)** ITS2 rDNA ML phylogenetic tree of the *Reticulata* group showing the phylogenetic relationship of ARK-S11-19 and ARK-S12-19 with other *C. reticulata*. For both phylogenetic trees, the best model was K2+I calculated by MEGAX 10.1.8. Numbers next to branches indicate statistical support value [ML bootstraps (1000 replicates)/Bayesian posterior probabilities]. The bootstrap support values above 70% and Bayesian posterior probabilities above 0.90 are shown. Thick lines indicate the branches with full statistical support (ML/BI:100/1.00).

The cells of isolates ARK-S11-19 and ARK-S12-19 were both ellipsoid-shaped, 12–13 μm in length and 6–7 μm in width (Fig. 3F and G), having a prominent hemispherical shaped papilla (Fig. 2 f6 and g6) and a cup-shaped chloroplast without a pyrenoid (Fig. 2 f1, f2, g1, g2 and Fig. S3 f, g). The eyespot was in the anterior 1/2–1/3 of the cell (Fig. 2 f1 and g1) and two contractile vacuoles were located on anterior side (Fig. 2 f5 and g5). The 18S rRNA gene sequence of ARK-S11-19 and ARK-S12-19 (1603 and 1631 bp long, respectively), and the 5.8S rDNA partial + ITS2 + 28S rDNA partial sequences of both isolates (404 bp long each) were identical to the type species *C. reticulata* CCAP 11/110 ( = CCCryo 213–05; SAG 29.83; UTEX 1970), a snow alga isolated from Todd lake, Oregon, USA. The phylogeny based on the 18S rRNA gene sequences using the ML method showed that both isolates belong to core *Chloromonas*, located within a subclade containing the type species and corresponding only to the *C. reticulata* clade (Pröschold et al. 2001) within clade 1 (Hoham et al. 2002) (Fig. 6A). Further phylogenetic analysis based on the ITS2 rDNA provided higher resolution and both isolates were clustered together with *C. reticulata*, within a well-supported (ML/BI: 99/0.91) subclade of the *Reticulata* group (Matsuzaki et al. 2012, Fig. 6B). The biogeographical location and habitat of selected *Chloromonas* strains (shown in Fig. 6A and B) is presented in Fig. S7, showing a multi-continental distribution of *C. reticulata* species.

Microscopic analysis revealed that the vegetative cells of ARK-S08-19 are round or ellipsoidal in shape, 10–20 μm in length and 7–8 μm in width, have a small hemispherical shaped anterior papilla (Fig. 2 e1), a nucleus and asteroid chloroplast with centrally located pyrenoid (Fig. 2 e1, e2, e8 and Fig. S1e) and two contractile vacuoles on the anterior side (Fig. 2e7). The vegetative cells have 12–14 μm long pairs of cilia and the eyespots are located on the anterior 1/3 of the cell (Fig. 2 e1 and e6). The non-motile cells of ARK-S08-19 are oval or spherical, up to 28 μm in diameter with pyrenoid (Fig. S8). The formation of two to sixteen zoospore cells within the parental cell wall was observed. Using the 18S rRNA gene sequence phylogeny, isolate ARK-S08-19 was located in a clade together with *C. augustae* within clade 1 (Hoham et al. 2002, Fig. 6A). Based on available GenBank data, the 18S rRNA gene sequence of ARK-S08-19 was most closely related to *C. augustae* SAG 13.89 isolated from a salt marsh pool in the United Kingdom, with 13 bp difference out of 1687 bp (99.23% similarity), followed by *C. augustae* SAG 5.73 (99.17% similarity), a freshwater alga isolated from the former Czechoslovakia, differing in 14 bp out of 1687 bp (99.17% similarity). The closest and only BLASTn hit of the ITS2 rDNA sequence of ARK-S08-19 against the NCBI database was *C. augustae* SAG 5.73, yet it differed by 32 bp out of 136 bp of aligned sequence (40% query cover, 76.5% similarity). Further ITS2 rRNA sequence-structure analysis showed that the alignable sequences were mainly found in helix II and near the 5’ side of helix III (Fig. S9).

### *Xanthonema*-like species

**Figure 7.**
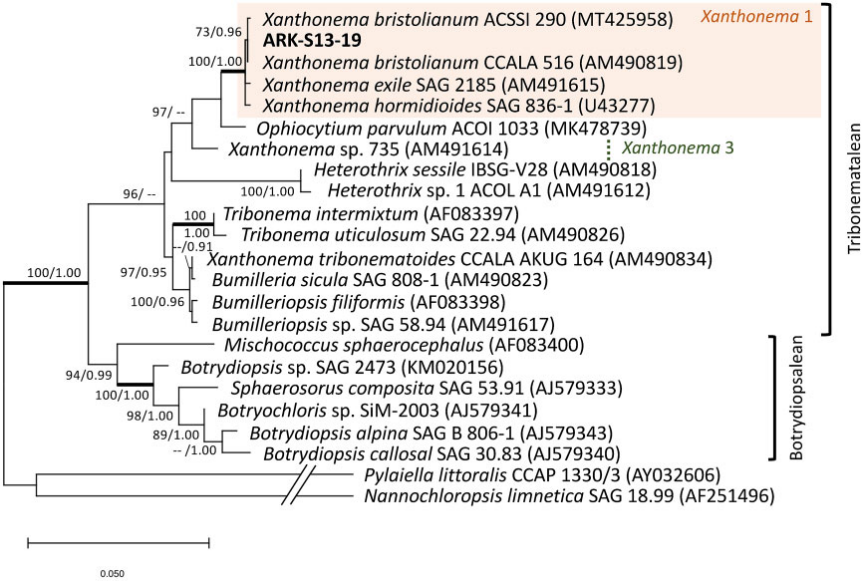
A phylogenetic tree based on the alignment of 18S rRNA gene sequences using the ML method for *Xanthonema*, the new isolate (sequence) is shown in bold. The best model was TN93 + G +I calculated by MEGAX 10.1.8. Numbers next to branches indicate statistical support values [ML bootstraps (1000 replicates)/Bayesian posterior probabilities]. The bootstrap support values above 70% and Bayesian posterior probabilities above 0.9 are shown. Thick lines indicate the branches with full statistical support (ML/BI:100/1.00). *Pylaiella littoralis* CCAP 1330/3 (AY032606) and *Nannochloropsis limnetica* SAG 18.99 (AF251496) serve as the outgroup.

The ARK-S13-19 cells were oblong with rounded corners, ∼8.4 μm in length 5.5 μm in width (Fig. 2 h1 to h5). The cells usually form unbranched filaments consisting of 2 to 30 cells, but it can sometimes be unicellular (Fig. 2 h4), having four to 12 plate-like chloroplasts (Fig. S3h). Eyespots were occasionally visible in some cells (Fig. 2h3 and h5) indicating the development of motile cells. Phylogenetic analysis of the 18S rRNA gene sequence using ML methods showed that ARK-S13-19 belongs to the fully statistically supported *Xanthonema* 1 clade (ML/BI:100/1.00) within the Tribonematales (Fig. 7) (Maistro et al. 2009). The 18S rRNA gene sequence of ARK-S13-19 (1708 bp long) was identical to *X. bristolianum* CCALA 516 isolated from snow in Belianske Tatras, Slovakia. The closely related strain of *X. bristolianum* ACSSI 290 was isolated from soil in Russia.

### Growth rate

**Figure 8.**
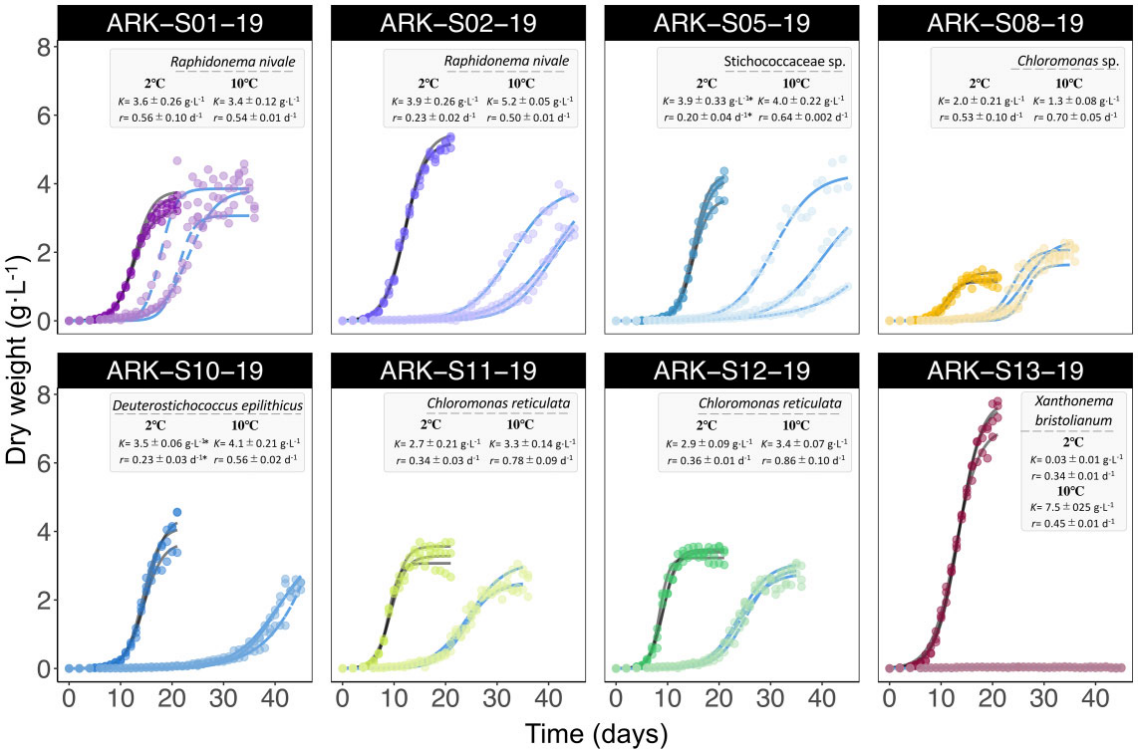
Growth patterns of eight microalgal isolates at 2°C and 10°C (Experiment 1). The carrying capacity (maximum cell mass production, *K*, g·L^−1^) and the intrinsic growth rate (*r*, d^−1^) are shown in the gray box. Mean values (±standard error) of triplicates per isolate are given in the table. The lines show the predicted growth models at 2°C (blue dashed line) and at 10°C (grey line). The coloured points indicate the measured biomass concentrations over 20–45 days (*n =* 3), where lighter coloured points for each isolate indicates the measurements at 2°C. The asterisks indicate the mean values (± standard error) of duplicates per isolate due to an outlier that did not reach the stationary phase.

The growth patterns of the eight isolates at 2°C and 10°C were modelled (Equation 1) and the results are presented in Fig. 8 and Table S3. At 10°C, the growth rate *r* varied from 0.45 ±0.01 to 0.86 ±0.10 d^−1^ amongst all isolates, with the highest growth rates recorded in *C. reticulata* ARK-S11-19 and ARK-S12-19. *X. bristolianum* ARK-S13-19 exhibited the highest carrying capacity (maximum cell mass production, *K*) reaching 7.5 ±0.25 g·L^−1^ whilst the lowest maximum cell production was achieved by *Chloromonas* sp. ARK-S08-19 with a *K* estimate of 1.3 ±0.08 g·L^−1^. At lower 2°C, close to the temperature of melting snow, all strains exhibited an extended lag phase and the growth rate of seven strains was substantially reduced compared to growth at 10°C. At 2°C maximum growth rates ranged from 0.20 ±0.04 to 0.56 ±0.10 d^−1^, with *R. nivale* ARK-S01-19 exhibiting the highest specific growth rate amongst the isolates, comparable to that obtained at 10°C. In contrast, the related isolate *R. nivale* ARK-S02-19 recorded protracted and substantially reduced growth rates of 0.23 ±0.02 d^−1^ at 2°C. *C. reticulata* ARK-S11-19 and ARK-S12-19 also recorded an extended lag phase, but eventually established intermediate growth rates of 0.34 ±0.03 and 0.36 ±0.01 d^−1^, respectively. At 2°C *X. bristolianum* ARK-S13-19 showed minimal growth over the whole cultivation period. Interestingly, ARK-S08-19 reached a higher cell density (*K*) of 2.0 ±0.21 g·L^−1^ at 2°C than that of 1.3 ±0.08 g·L^−1^ obtained at 10°C, and its growth rate at low temperature was not suppressed to the same extent as most of the other strains.

### Fatty-acid profiles

The total fatty acid (TFA) contents of each isolate cultivated under standardized conditions with excess nutrients (Experiment 2) ranged from 54.3 to 93.0 mg·g^−1^ DW, with total polyunsaturated fatty acids accounting for between 57.3% and 70.4% of TFA (Table 2). The fatty acid profiles of both *R. nivale* isolates, ARK-S01-19 and ARK-S02-19, were distinguished by substantial amounts of C18:3n-3, accounting for 25.2 and 19.1% of TFAs (15.1 and 10.6 mg·g^−1^ DW) respectively, and C20:5n-3 (eicosapentaenoic acid, EPA) amounting to 19.7 and 16.6% of TFAs (11.8 and 9.2 mg·g^−1^ DW), respectively. C16:0 was also found at intermediate levels (11.2 and 12.5% TFAs, 6.7 and 6.9 mg·g^−1^ DW, respectively). The two *R. nivale* strains differed slightly, with ARK-S01-19 containing additional trace amounts of C22:0, C20:4n-3, and C22:6n-3 (docosahexaenoic acid; DHA), which were absent in ARK-S02-19 (Table 2).

**Table 2.** TFA and fatty acid composition of eight isolates in nutrient-replete conditions (Experiment 2) (mg.g^−1^ DW). Mean values (±SD) of triplicates per isolate are given in the table.

	*R. nivale* ARK-S01-19	*R. nivale* ARK-S02-19	*Stichococcaceae* sp. ARK-S05-19	Chloromonas sp. ARK-S08-19	*D. epilithicus* ARK-S10-19	*C. reticulata* ARK-S11-19	*C. reticulata* ARK-S12-19	*X. bristolianum* ARK-S13-19
C14:0	2.9 ± 0.05	2.6 ± 0.5	5.1 ± 0.1	2.2 ± 0.1	2.8 ± 0.1	2.2 ± 0.3	2.4 ± 0.1	9.9 ± 1.2
C16:0	6.7 ± 0.7	6.9 ± 1.1	4.6 ± 0.4	7.7 ± 0.2	3.9 ± 0.6	10.1 ± 0.7	9.8 ± 0.5	4.5 ± 0.7
C16:1n-7	1.2 ± 0.1	2.5 ± 0.5	1.8 ± 0.1	1.7 ± 0.02	1.0 ± 0.03	1.0 ± 0.1	0.7 ± 0.5	12.1 ± 1.6
3t-16:1	1.6 ± 0.1	1.5 ± 0.3	2.6 ± 0.1	1.4 ± 0.03	1.5 ± 0.1	1.3 ± 0.1	1.4 ± 0.1	3.3 ± 0.4
C16:2n-6	–	–	–	–	–	–	–	0.5 ± 0.1
C16:2n-4	–	–	–	–	–	–	–	5.7 ± 0.7
C16:3n-4	–	–	–	–	–	–	–	9.3 ± 1.2
C16:3n-3	1.8 ± 0.1	1.1 ± 0.1	12.2 ± 0.3	3.3 ± 0.1	6.5 ± 0.5	4.8 ± 0.3	4.6 ± 0.5	–
C16:4n-3	4.3 ± 0.1	4.8 ± 1.1	–	13.6 ± 0.3	–	16.6 ± 0.9	16.3 ± 1.0	–
C18:0	–	–	–	–	–	–	–	–
C18:1n-9	3.2 ± 0.2	2.9 ± 0.2	–	13.2 ± 0.9	2.3 ± 0.4	7.8 ± 0.7	8.4 ± 01.4	–
C18:1n-7	4.3 ± 0.2	5.9 ± 1.0	7.3 ± 0.2	10.5 ± 0.2	3.6 ± 0.1	9.3 ± 0.6	9.0 ± 0.6	–
C18:2n-6	0.6 ± 0.1	1.9 ± 0.2	2.3 ± 0.1	1.3 ± 0.05	2.4 ± 0.4	0.7 ± 0.05	0.5 ± 0.2	2.0 ± 0.3
NMI-18:3	–	–	–	–	–	–	–	0.5 ± 0.1
C18:3n-3	15.1 ± 1.4	10.6 ± 1.9	26.6 ± 0.7	38.2 ± 0.5	17.7 ± 1.0	37.3 ± 2.3	36.1 ± 0.31	–
C18:3n-4	–	–	–	–	–	–	–	1.6 ± 0.2
C18:4n-4	–	–	–	–	–	–	–	4.0 ± 0.6
NMI-18:4	–	–	–	–	–	–	–	2.8 ± 0.3
C18:4n-3	2.0 ± 0.1	4.1 ± 0.8	–	–	1.3 ± 0.1	–	–	–
C20:0	–	–	–	–	–	–	–	–
C20:1n-9	0.7 ± 0.03	1.2 ± 0.1	–	–	–	–	–	–
C20:3n-6	–	–	–	–	–	–	–	–
C20:4n-6	–	–	2.5 ± 0.1	–	5.0 ± 0.9	–	–	3.1 ± 1.1
C20:4n-3	1.7 ± 0.1	–	–	–	1.6 ± 0.1	–	–	–
C20:5n-3	11.8 ± 0.3	9.2 ± 1.7	5.0 ± 0.2	–	3.7 ± 0.2	–	–	27.3 ± 3.3
C22:0	0.1 ± 0.3	–	–	–	0.8 ± 0.1	–	–	–
C24:0	0.4 ± 0.2	0.1 ± 0.1	0.6 ± 0.1	–	0.3 ± 0.1	–	–	–
C22:6n-3	1.6 ± 2.0	–	–	–	–	–	–	2.5 ± 0.3
Omega-3	38.2 ± 0.9	29.7 ± 5.4	43.8 ± 1.1	55.2 ± 0.9	30.8 ± 1.8	58.7 ± 3.4	57.1 ± 4.5	30.3 ± 3.7
Omega-6	0.6 ± 0.1	1.9 ± 0.2	4.7 ± 0.1	1.3 ± 0.05	7.4 ± 1.0	0.7 ± 0.04	0.5 ± 0.2	5.6 ± 1.3
Omega-4	–	–	–	–	–	–	–	23.5 ± 3.1
SFA	10.1 ± 0.3	9.5 ± 1.5	10.1 ± 0.4	9.6 ± 0.4	7.8 ± 0.7	11.8 ± 0.9	11.9 ± 0.7	14.2 ± 1.9
MUFA	11.0 ± 0.3	14.0 ± 1.9	11.7 ± 0.3	26.9 ± 1.0	8.3 ± 0.4	19.4 ± 1.5	19.5 ± 1.5	15.4 ± 2.0
PUFA	38.8 ± 0.9	31.7 ± 5.5	48.6 ± 1.1	56.4 ± 0.8	38.2 ± 1.9	59.8 ± 3.5	57.6 ± 4.6	59.4 ± 7.4
TFAs	59.9 ± 4.1	55.1 ± 3.2	70.4 ± 7.4	93.0 ± 11.3	54.3 ± 4.2	91.1 ± 11.1	88.9 ± 10.8	89.0 ± 6.9

Stichococcaceae sp. ARK-S05-19 and *D. epilithicus* ARK-S10-19 were characterized by high amounts of C18:3n-3, accounting for 37.7% and 32.5% of TFAs (26.6 and 17.7 mg·g^−1^ DW), respectively. C16:3n-3 was also abundant, accounting for 17.4% and 11.9% TFAs (12.2 and 6.5 mg·g^−1^ DW), respectively.

The FAME profiles of *Chloromonas* isolates ARK-S08-19, ARK-S11-19 and ARK-S12-19 revealed the dominance of C18:3n-3, which accounted for 40.6%–41.1% of TFAs, with additional fatty acids including C16:4n-3, C16:0, C18:1n-7, and C18:1n-9. Although profiles were broadly comparable, some differences in fatty acid proportions were observed, for example *Chloromonas* sp. ARK-S08-19 had higher proportions of C18:1n-9 (14.2% TFAs) than *C. reticulata* ARK-S11-19 and ARK-S12-19 (8.6 and 9.4% TFAs, respectively).

In contrast to the other isolates investigated in this study, *X. bristolianum* was characterized by the presence of the omega-4 fatty acids C16:2n-4, C16:3n-4, C18:3n-4, and C18:4n-4. In addition, *X. bristolianum* contained unusual (non-methylene interrupted) NMI-18:3 and NMI-18:4 fatty-acids (Fig. S10), of which the mass spectrum was not included in one of the best-known existing libraries of C18 fatty acids (Christie 2021). The most abundant FA in *X. bristolianum* was EPA (30.7% TFAs) followed by C16:1n-7 and C14:0.

FAME profiles were also examined in the algal samples collected from the stationary phase of the growth rate experiment at 10°C (Experiment 1, Fig. S11). The amount of TFAs was higher in all isolates (155.3–411.6 mg·g^−1^ DW) than that in Experiment 2 and the FA profile of each isolate was characterized by accumulation of a few fatty-acids. Substantial proportions of C18:1n-9 were observed in *Raphidonema, Stichococcus*, and *Chloromonas* strains accounting for the majority (48.7–57.2%) of TFAs. *Xanthonema* sp. ARK-S13-19 accumulated primarily C16:1n-7 reaching 50.2% of TFAs, followed by C16:0 (17.3% TFAs), C14:0 (16.8% TFAs), and only a small amount of C18:1n-9.

### LD dynamics in *C. reticulata*

**Figure 9.**
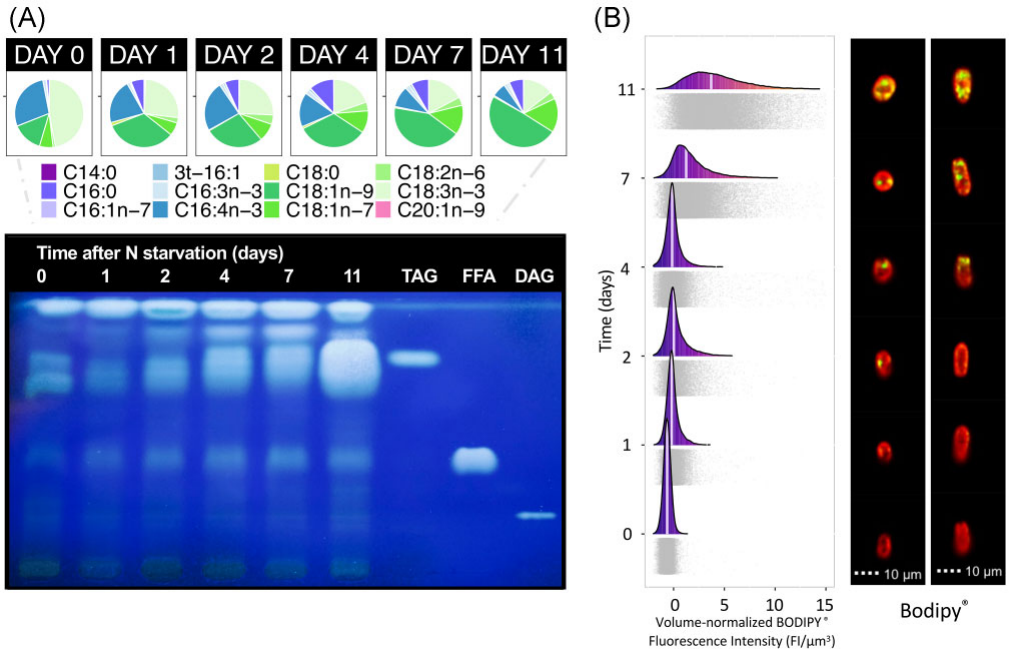
Neutral lipid accumulation in *C. reticulata* ARK-S12-19 under nitrogen starvation (Experiment 3). **(A)** Analysis of TAG accumulation (bottom left) and the average TAG fatty-acid composition (top left) from *n* = 3 replicates in *C. reticulata*. 3t-16 : 1 indicates 3-trans-hexadecenoic acid. TAG content is determined by high performance thin-layer chromatography (HPTLC) together with standards (TAG; triacylglycerol, FFA; free fatty acids, DAG; diacylglycerol). The plate is labelled with Primuline and visualized under 254 nm UV light. (B) Flow-cytometric analysis of cell biovolume-normalized Bodipy^®^ fluorescence over 11 days (*n* = 3). The white vertical lines indicate the median of the frequency and the grey shaded points indicate individual cell data. Multispectral images show the accumulation of LDs in the cells. The Bodipy^®^ fluorescence (green) and chlorophyll autofluorescence (red) are shown. R, region: R2; round shaped cells, R4; oval shaped cells.

We selected cosmopolitan *C. reticulata* ARK-S12-19 as a model to study LD formation under N-starvation, using both chemical lipid analysis by GC-FID and image-based labelling with imaging flow cytometry (Experiment 3). Over the 11-day experimental period, neutral and polar lipids were separated by solid-phase extraction (Fig. S12), and TAG was further separated from other neutral lipids by thin layer chromatography (TLC, Fig. 9A). Polar lipids peaked at 127.6 mg·g^−1^ DW at day 7, and decreased to 75.0 mg·g^−1^ DW after 11 days of N removal, indicating limited membrane lipid degradation towards the end of the experiment (Fig. S12). During the same period, total neutral lipids increased from 14.2 to 208.7 mg·g^−1^ DW. TLC analysis revealed that C16:4n-3 and C18:3n-3 fatty acids dominated TAG at the start of the experiment, but by day 11 fatty acids C18:1n-9 and C18:1n-7 became the most abundant (Fig. 9A). Using imaging flow cytometry (IFC) we were able to distinguish different cell types including oval shaped bicilliates (27.5% ±5.2%; R4) and cells lacking cilia (35.6% ±6.5%; R3) (Fig. S13). Bodipy^®^ probe fluorescence showed an increase in neutral lipids over time, which was confirmed by visualizing the size, position and number of LDs within the cells (Fig. 9B). As variation in cell size could influence the quantitation of neutral lipids by IFC, we tested whether the normalization of Bodipy^®^ fluorescence to the image bright field area (µm^2^) and biovolume of individual cells (µm^3^) improved the correlation between IFC (Bodipy^®^) and GC quantification (SPE and TLC) of fatty acids (Fig. S14 and S15). Normalizing the Bodipy^®^ 493/503 fluorescence to either 2D cell image area or cell biovolume resulted in improved correlation with TFAs, neutral lipids, and TAG compared to non-normalized Bodipy^®^ fluorescence (Fig. S15). The correlation coefficients were slightly higher for neutral lipids vs Bodipy^®^ (*r^2^* = 0.97) than TAG vs Bodipy^®^ (*r^2^* = 0.95) (Fig. S15).

## Discussion

Snow-algae communities harbour substantial biodiversity, yet their phylogeny and global distribution are not well understood. This study is the first to isolate and cultivate snow algae from mainland Norway. Our use of relatively conserved (18S rRNA gene) and highly variable (ITS2 rDNA) genetic markers combined with microscopy and lipid profiling will assist detection of these species widely in environmental samples.

### Phylogeny using molecular markers

*Raphidonema* is a cosmopolitan, globally dispersed genus (Segawa et al. 2018) commonly found in alpine or glacier ecosystems (Leya et al. 2009, Yakimovich et al. 2021, Novis et al. 2023, Segawa et al. 2023). Using morphology alone, *Raphidonema* species are difficult to distinguish from other closely related Trebouxiophycean genera, especially *Koliella*, due to their similar cell appearance combined with phenotypic plasticity under different conditions (Hoham 1973, Stibal and Elster 2005). The phylogeny of *Koliella* and *Raphidonema* was recently resolved with molecular markers, where strains designated as *Raphidonema* or *Koliella* were clearly separated based on *rcb*L sequences (Yakimovich et al. 2021). The authors further revised *Raphidonema* using the ITS2 rRNA sequence-structure and proposed five species within the genus. Our analyses showed that the 18S rRNA gene was not adequate in delineating the isolates at species level, but ITS2 rRNA sequence-structure analysis provided high resolution and places ARK-S01-19 and ARK-S02-19 as different strains of *R. nivale*. Most of the existing *Raphidonema* cultures in collections were isolated from snow and glacier habitats (CCCryo culture collection, the Culture Collection of Algae at the University of Texas at Austin, UTEX), but Stibal and Elster (2005) reasonably suggest that *Raphidonema* is a soil-dweller transported onto snow by wind or meltwater. Although *R. nivale* has not yet been cultured from soil, closely related species, including *R. pyrenoidifera* and *R. monicae* have been isolated from this habitat (Yakimovich et al. 2021). Further investigation of summer Arctic and Alpine soil communities (e.g. by amplicon sequencing) could improve our understanding of the lifestyle and global distribution of *Raphidonema*.

*Stichococcus*-like strains belonging to the Prasiola clade are widely distributed in various habitats, from tropical to polar regions, freshwater to marine, and from hot acidic springs to snow and cryoconite hole environments (Butcher 1952, Stibal et al. 2006, Hodač et al. 2016). They often change their morphotype, making it difficult to designate strains at genus and species levels without sequence information (Pröschold and Darienko 2020). Recently, the genus *Stichococcus* was divided into seven lineages using a multigene approach by Pröschold and Darienko (2020). According to our ITS2 rRNA sequence-structure based phylogeny, isolate ARK-S10-19 clearly belongs to one of the seven lineages under *Deuterostichococcus* designated as *D. epilithicus* (Pröschold and Darienko 2020). However, ARK-S05-19 formed a distinct and unique clade with the isolate Prasiolales sp. S2RM26 as an undescribed sister group to *Stichococcus* and *Desmococcus*. According to Coleman (2003), the closely related species could be delineated by the presence of CBCs in the conserved region of the ITS2 rRNA secondary structure, as it correlates with the separation of biological species. Although a CBC in helix I of ITS2 rRNA between ARK-S05-19 and S2RM26 was found in a position assumed to be outside the conserved region as defined by Pröschold and Darienko (2020), the two algae were clearly phylogenetically distinct (Fig. 4), suggesting that this clade may contain hidden species diversity. ARK-S05-19 displayed *Stichococcus*-like morphology rather than that of *Desmococcus*, which can be easily distinguished by the unique characteristics of *Desmococcus* including formation of package-like structures with branched filaments and production of akinetes with ornamented cell walls (Broady and Ingerfeld 1993). Unfortunately, morphological comparison with Prasiolales sp. S2RM26 could not be performed, and ARK-S05-19 is tentatively classified as Stichococcaceae sp. Further morphological comparison with closely related strains and sequencing of 18S rRNA gene and *rbcL* genes within the clade could in theory facilitate establishment of new species and improve our understanding of the biogeography and ecophysiology of Stichococcaceae and their appearance in melting snowfields worldwide.

The most commonly found snow algae are typically members of the *Chloromonadinia* clade within the Volvocales (Barcytė et al. 2018a, Hoham and Remias 2020). The *Chloromonadinia* originally comprised solely the genus *Chloromonas* based on *C. reticulata* (Gobi) as the type species, but further investigation led to the discovery of new clades, and the genera *Chlainomonas* (Christen 1959, Novis et al. 2008), *Gloeomonas* (Klebs 1886, Nozaki et al. 2010), *Ostravamonas* (Hoham and Remias 2020, Susanti et al. 2020), and *Ixipapillifera* (Nakada et al. 2016) are currently placed within the same phylogroup. Our sequence analyses show that ARK-S11-19 and ARK-S12-19 are identical to the type species *C. reticulata* Gobi CCAP 11/110 ( = CCCryo 213–05; SAG 29.83; UTEX 1970) placed in the *Reticulata* group (Matsuzaki et al. 2012), within the core *Chloromonas* (part of clade 1) that were suggested to be a true *Chloromonas* genus by Barcytė et al. (2018a). Both isolates also exhibited the key morphological features of *C. reticulata* which distinguishes them from three closely related species in the *Reticulata* clade (Matsuzaki et al. 2012). Whilst *C. reticulata* was previously evaluated as a robust cryotolerant mesophile (CCALA 970 in Lukeš et al. 2014 was isolated from red snow and belongs to *C. reticulata*, Barcytė et al. 2018b), it likely does not form snow-blooms, though it is commonly found in snow samples using culture-dependent techniques (Novakovskaya et al. 2018). In contrast, clade 2 encompasses psychrophilic microorganisms forming snow blooms, supported by Sanger sequences from field-collected cells in blooms that are conspecific with those obtained from laboratory strains (Matsuzaki et al. 2019). Still, *C. reticulata* has been isolated from multiple continents; CCAP 11/110 ( = CCCryo 213–05; SAG 29.83; UTEX 1970) from snow in the USA, *C. reticulata* SAG 32.86 from moss in Antarctica, CCCryo 338–08 from snow in France, SAG 26.90 a Norwegian lake and SYKOA Ch-054–11 from snow in Russia (Fig. S7). Our analysis further supports the ecological versatility and cosmopolitan distribution of *C. reticulata* in cold habitats, including snow in northern Norway.

On the other hand, the 18S rRNA gene sequence designated ARK-S08-19 as *Chloromonas* sp. and placed it within a clade containing *C. augustae*, yet its partial 5.8S rDNA + ITS2 rDNA + partial 28S rDNA had only one BLASTn hit to *C. augustae* SAG 5.73 with very low query cover (40%). Such low coverage of ITS2 rDNA sequences together with relatively low similarity of the 18S rRNA gene suggests that ARK-S08-19 may be an independent novel species. Yet, morphologically, the vegetative cells resemble those of *C. augustae* (Skuja 1943, Pröschold et al. 2001). *Chloromonas* augustae has been isolated from freshwater habitats including paddy soil, swamp, and salt marsh pools (Pröschold et al. 2001), though there are no previous reports from melting snow. Sequencing the ITS2 rDNA of related strains and the use of additional markers such as *rbc*L genes could provide more robust species-level identification, whilst ultrastructural analysis may also aid characterization of ARK-S08-19.

Lastly, the genus *Xanthonema* comprise freshwater algae that have been found in soil, snow and freshwaters from alpine, polar, and temperate environments (Broady et al. 1997, Andersen 2004, Broady 2005). The most recent taxonomic assessment of Tribonematalean algae was based on *rbc*L and 18S rRNA gene sequences, where *Xanthonema* clade 1 was defined as the genus *Xanthonema*, and clade 2 and 3 were defined as new genera awaiting taxonomic revision (Maistro et al. 2009, Rybalka et al. 2009). Our analyses showed that ARK-S13-19 is *X. bristolianum* and identical to CCALA 516, placed within *Xanthonema* clade 1 (Rybalka et al. 2009). Molecular evidence was supported by the cell and chloroplast morphologies of ARK-S13-19, which resembled that of *X. bristolianum* CCALA 516 as described by Pascher (1939), although we occasionally observed eyespots in some cells that were not mentioned in the original description. Considering the reports of *Xanthonema* in colder habitats including soil and snow (Elster et al. 1999, Rybalka et al. 2009, Schulz et al. 2016, Borchhardt et al. 2017a,b), *X. bristolianum* is probably widely dispersed across the globe, and its absence in snow algae communities in previous metabarcoding studies (Segawa et al. 2018, Lutz et al. 2019) suggests that *Xanthonema* is an opportunistic soil alga rather than a typical snow-inhabiting species.

A limitation of our methodology is that the most abundant red-pigmented cells in the snow samples may not be readily culturable. Researchers have reported only limited success in recovering carotenoid-rich encysted red stages into green, viable cultures (e.g. Procházková et al. 2018a,b). However, Raymond et al. (2022) showed that orange-coloured cells recognized as *Sanguina aurantia* in field samples were conspecific with their isolates of green biciliated cells that are characteristic of Volvocine algae. Their cultures could only be kept in liquid medium, implying that related *Sanguina* sp. and presumably similar algae causing red snow may have specific cultivation requirements. Similarly, Procházková et al. (2019b) obtained cultivable green cells, which were conspecific with field collected orange cysts of *Chloromonas hindakii*. The authors allowed the cysts to recover and produce a motile, ciliated cell culture in deionized water under laboratory conditions by simulating the diurnal temperature regime of melting snow. *Chloromonas hindakii* has been reported to propagate faster in low nutrient medium (0.6 N BBM) (CCCryo culture collection), and the use of standard media in our study may have selected strains that are better adapted to high-nutrient conditions. Expanding the range of isolation and cultivation conditions could potentially increase the number of unique strains obtained, whilst comparing isolates to whole community level metabarcode (amplicon) sequences from the same red snow microbiomes may help to establish the relative contribution of our isolates to in-situ snow algae communities.

### Snow-algae dispersal, community construction, and physiological traits

Snow algae communities in northern Europe have not been extensively studied and until recently little was known about their distribution and how their constituent species compared with visibly similar red-snow microbiomes around the world. Our isolates include cosmopolitan species together with new, unique strains that are potentially endemic to the region. Assuming that microbial dispersal is not a major barrier to initial colonization, what shapes snow-algae microbiomes in different locations? Recent data implicate a multitude of factors driving variation in emergent communities that, for example, vary with altitude in the same region (Yakimovich et al. 2020, Stewart et al. 2021). The interplay between temperature, underlying soil structure, and snowmelt dynamics thus likely mediate the annual propagation of alternative species and community structures at different sites. Especially, the growth rate of microbes is a fundamental life history trait that determines their ecological success, the outcome of competitive interactions and ultimately the composition of natural microbial communities and their biogeochemical functions (Pianka 1970, Dorodnikov et al. 2009, Brown et al. 2016). At 10°C the growth rates of our isolates varied two-fold from 0.45 to 0.86 d^−1^. *Chloromonas reticulata* ARK-S11-19 and ARK-S12-19 presented the highest specific growth rates, whilst novel *Chloromonas* sp. ARK-S08-19 was characterized by substantially lower growth rates and carrying capacity, reflecting acute competitive differences between related strains. At lower 2°C temperatures close to that of melting snow, the growth rates of all strains except *R. nivale* ARK-S01-19 were suppressed, and differences between strains were more conspicuous. The higher growth rate of *R. nivale* strain ARK-S01-19 compared with ARK-S02-19 at 2°C highlights how ecotypic variation and selection on thermal tolerance may reshape snow algae communities. Although they are the same species, the closest relative of ARK-S01-19 was isolated from Svalbard (77°N), suggesting that ARK-S01-19 may have acquired physiological adaptation to lower temperatures compared with ARK-S02-19. *Chloromonas* isolates ARK-S08-19, ARK-S11-19, ARK-S12-19 exhibited strong growth rates (0.34–0.53 d^−1^) and reached consistent, high cell densities at 2°C, supporting the widespread global distribution of this genus in melting snow. The ephemeral and dynamic nature of melting snowfields implies that opportunistic *r*-strategists with higher growth rates, and possibly high motility, should prevail in these conditions. Nevertheless, snow and ice are multi-stress environments characterized by high light, freeze-thaw cycles, and limited nutrients that may impose additional selective constraints on the microbes capable of surviving there that may explain the coexistence and succession of diverse species (Blagodatskaya et al. 2007, Krug et al. 2020, Tucker and Brown 2022). Further, in-situ growth rates and the recruitment of snow microbial communities from the soil are probably further curbed by access to light and nutrients.

Our data show that fatty acid profiles are species and culture age specific. Under standard high-nutrient conditions (Experiment 2) the Chlorophyta *Raphidonema*, Stichococcaceae, and *Chloromonas* were characterized by abundant n-3 PUFAs, predominantly C18:3n-3, with increases in C18:1n-9 in the stationary phase. C18:3n-3 is commonly found in plastidial membrane lipids monogalactosyldiacylgelycerol, digalactosyldiacylglycerol, sulfoquinovosyldiacylglycerol, and phosphatidyl-glycerol (Li-Beisson et al. 2015), whilst C18:1n-9 is known to accumulate in TAG under growth limiting conditions in Chlorophyta (Solovchenko 2012, Andeden et al. 2021, Wan Afifudeen et al. 2021). C18:1n-9 has been reported as a dominant fatty acid in many isolated snow algae strains in N-deprived or stationary phase conditions, including *Raphidonema* and *Chloromonas* (Spijkerman et al. 2012, Hulatt et al. 2017, Suzuki et al. 2019), as well as in field samples of red snow composed of *Sanguina nivaloides* (Procházková et al. 2019a) and other red, orange, or green snow (Bidigare et al. 1993, Spijkerman et al. 2012, Davey et al. 2019). On the other hand, monospecific snow algae blooms caused by *C. kaweckae* (green, Procházková et al. 2022), *C. nivalis* subsp.tatrae, subsp. (brownish-red, Procházková et al. 2018a), *Chloromonas krienitzii* (orange, Procházková et al. 2020), *C. hindakii* (orange, Procházková et al. 2019b), *Chlainomonas* sp. (red, Procházková et al. 2018b) contained largely C18:3n-3 fatty acids rather than C18:1n-9. The fatty acid C16:4n-3 was also a major feature of genus *Chloromonas* and secondly *Raphidonema*, which is found exclusively in plastidal membrane monogalactorsyldiacylgelycerol in *Chlamydomonas reinhardtii*, and occasionally present in the Trebouxiophyceae (Lang et al. 2011). Both *Raphidonema* strains produced moderate proportions of the long-chain PUFA C20:5n-3, which has previously been described amongst related strains from Svalbard (Spijkerman et al. 2012) and the Antarctic (Suzuki et al. 2019).

The fatty acid composition of *X. bristolianum* ARK-S13-19 (Ochrophyta) differed substantially from that of the Chlorophyta. *X. bristolianum* was characterized by a large proportion of C20:5n-3 under high-nutrient conditions, and by large amounts of C16:1n-7 in the stationary phase. The latter FA is not generally abundant in the Chlorophyta and has been detected only at low concentrations in red, orange, and green snow algal blooms in the field (Spijkerman et al. 2012, Davey et al. 2019). *X. bristolianum* was also characterized by multiple omega-4 fatty acids, including C16:2n-4 and C16:3n-4 which have previously been reported within the kingdom Chromista including the related species *Tribonema ultriculosum* (Chen et al. 2021), diatoms including Phaeodactylum tricornutum and Chaetoceros (Bacillariophyta) (Miller et al. 2014, Qiao et al. 2016), and Isochrysis aff. *galbana* (Haptophyta) (Huerlimann et al. 2014). Both C16:2n-4 and C16:3n-4 are reportedly located primarily in the thylakoid membrane lipid monogalactodiacylglycerol (Remize et al. 2020). However, to our knowledge the C18:3n-4, C18:4n-4, NMI-18:3 and NMI-18:4 fatty-acids found in ARK-S13-19 do not seem to have been reported in environmental samples nor other microalgae, and further lipidomic and genomic investigation may elucidate their biosynthetic pathways and functional roles.

Lipids can provide information on the physiological status of snow algae communities (Lutz et al. 2015). Davey et al. (2019) showed that red snow in Antarctica could be clearly distinguished from green snow by the elevated neutral lipids (TAGs) in red snow communities. TAGs are particularly relevant in snow-algae because LDs are produced under stress, especially the low-nutrient conditions often found in water films infiltrating melting snow crystals (Spijkerman et al. 2012). The fatty acid composition of TAG varies between species, reflecting the availability of alternative fatty acids and the activity of a range of acyltransferase enzymes (Xin et al. 2019). TAG in *C. reticulata* ARK-S12-19 under nitrogen starvation contained comparatively less saturated C16:0 fatty acids than related model alga *C. reinhardtii*, although the PUFA contents of *C. reticulata* (26%) was more comparable. PUFA-rich TAG has been reported in other algae and hypothesized to provide a reservoir for rapid reconstruction of membrane lipids, enabling cells to recover efficiently from environmental changes (Bigogno et al. 2002), although metabolic validation is needed. IFC analysis provided sensitive detection of the onset of LD development in *C. reticulata* and quantitatively supported neutral lipid/TAG inside single cells. Because the most abundant Chlamydomonadalean snow algae comprise multiple cell types, IFC-based analysis could be further extended for rapidly characterizing cell-cell heterogeneity and the ecophysiology of communities in nature.

## Conclusion

In this study, eight algae strains were isolated and characterized from red snow in northern Norway, adjacent to the Arctic Circle. Using molecular and morphological analyses the isolates were assigned to the species *R. nivale, D. epilithicus, C. reticulata*, and *X. bristolianum* as well as potentially novel species belonging to the Stichococcaceae family and *Chloromonas*. Cultivation revealed variation in growth rates and lipids, ecologically important traits that shape snow algae communities and their physiological properties in the natural environment. The accumulation of TAG in LDs was also observed in cosmopolitan *C. reticulata*, illustrating the adaptive metabolic changes that snow algae undergo to survive in harsh environments. The new cultures will provide further opportunities to explore the stress ecophysiology and molecular biology of snow algae in the laboratory.

## Supplementary Material

fiad057_Supplemental_File
