# Supplementary material for: Phylogeny and lipid profiles of snow-algae isolated from Norwegian red-snow microbiomes
Source: FEMS Microbiol Ecol. 2023 May 24;99(6):fiad057. doi: 10.1093/femsec/fiad057 (PMC10246834; doi:10.1093/femsec/fiad057)
Supplement: fiad057_Supplemental_File [file fiad057_supplemental_file.pdf]

Supplementary Information

Phylogeny and lipid profiles of snow-algae isolated from Norwegian red-snow microbiomes

Hirono Suzuki[1], Alexandre Détain[1], Youngjin Park[1], Viswanath Kiron[1], René H. Wijffels[1,2], Nathalie Leborgne-Castel[3], Lenka Procházková[4, 5], Chris J. Hulatt[1]*

Affiliations:

1. Faculty of Biosciences and Aquaculture, Nord University, Bodø, Norway
2. Bioprocess Engineering, AlgaePARC, Wageningen University, Wageningen, the Netherlands
3. Agroécologie, Institut Agro Dijon, CNRS, INRAE, Univ. Bourgogne Franche-Comté, Dijon, France
4. Department of Ecology, Faculty of Science, Charles University, Viničná, Prague, Czech Republic
5. Centre for Phycology, Institute of Botany of the Czech Academy of Sciences, Dukelská, Třeboň, Czech Republic

Correspondence: Chris J. Hulatt, Faculty of Biosciences and Aquaculture, Nord University, Bodø, Norway; *Phone*: (+47) 90098564; *Email*: christopher.j.hulatt@nord.no

MATERIALS AND METHODS

Fatty-acid analysis including extraction, isolation of lipids and methylation

For Experiment 2 and 3, total lipids were extracted from freeze-dried biomass using chloroform and methanol as described by Breuer *et al.* (2012). A bead mill (Precellys, Bertin Technologies, Montigny le Bretonneux, France) and 0.1 mm glass beads were used for cell lysis and lipids were recovered by addition of Tris buffer, followed by mixing with a vortex (10s) and centrifugation (3000 *g*, 5 min). The chloroform phase containing the crude lipid extract was transferred to new tubes and dried under a stream of N_2. For experiment 1, we directly extracted and derivatized lipids from liquid culture as described by Radakovits *et al.* (2012). In this method 1.0 ml of 0.04 g mL^{-1} KOH in 95% methanol was added directly to 0.5 ml of algal culture and heated in tightly sealed vials at 100°C (90min) for lipid saponification. Then 6% (v/v) of 12M HCl in methanol was added to the vials and incubated at 60°C for 12h. Fatty acid methyl esters (FAMEs) were extracted into 1.0 ml hexane.

For Experiment 3, neutral lipids and polar lipids were separated by solid-phase extraction (Sep-Pak 1 g silica cartridges, 6 mL, Waters, MA, USA). The lipid fractions were resuspended into a chloroform and methanol mix (2:1, v/v), and a standardized amount of sample equivalent to 1 mg DW of algae were transferred to new tubes and evaporated under a stream of N_2. To analyze triacylglycerol (TAG), the neutral lipid fraction was further separated with high performance thin layer chromatography (silica gel 60 F254 Premium Purity HPTLC glass plates 20 × 10 cm, Merck KgaA, Darmstadt, Germany). Lipids were resuspended in chloroform and methanol mix (2:1, v/v) and a standardized amount of sample equivalent to 1 mg DW were spotted onto plates using a microcapillary, and then the spots were dried under a stream of N_2. HPTLC plates were subsequently developed by a solvent mixture of hexane: diethyl ether: acetic acid (70:30:1). The developed TLC plates were sprayed with 0.01% (w/v) primuline in 80% (v/v) acetone and then the lipid bands were visualized under 254 nm UV light (Camag UV Cabinet 4, Camag, Muttenz, Switzerland). The bands were then scraped off and transferred to new tubes for methylation.

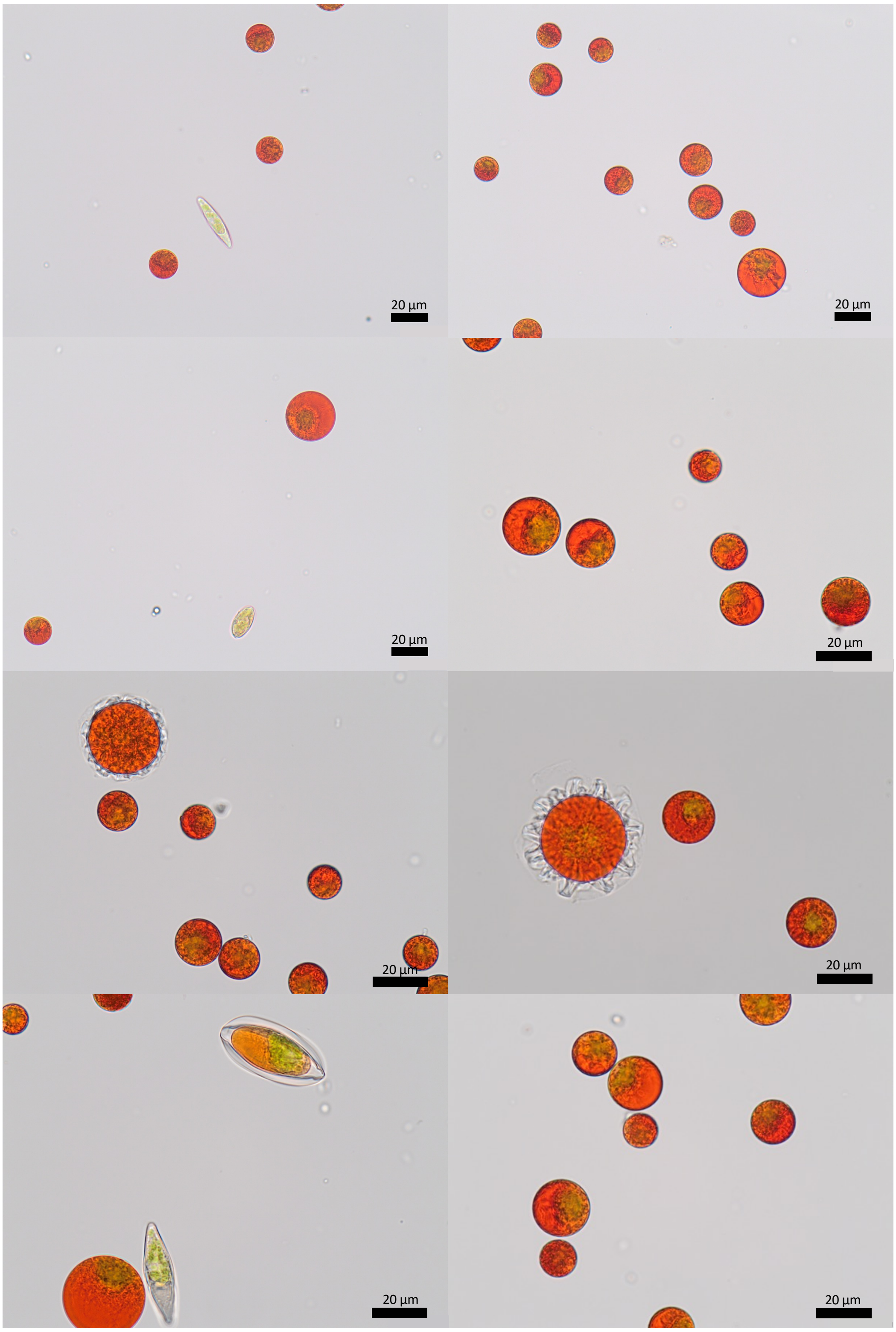

Supplementary Figure 1 (Fig. S1). Light microscopic pictures of the red snow samples from which we obtained the eight isolates. The samples were collected from Northern Norway adjacent to the arctic circle (66°45’03.9N 14°05’55.0E). Red pigmented cells dominated the samples. The dominant red cells morphologically resemble *Sanguina nivaloides*, whist scattered cells may be identified as *Chloromonas cf. nivalis* zygotes (cell with cell wall surface flanges) and *Scotiella cryophila* K1-like cysts (spindle shaped cell). Scale bars shown are 20 µm.

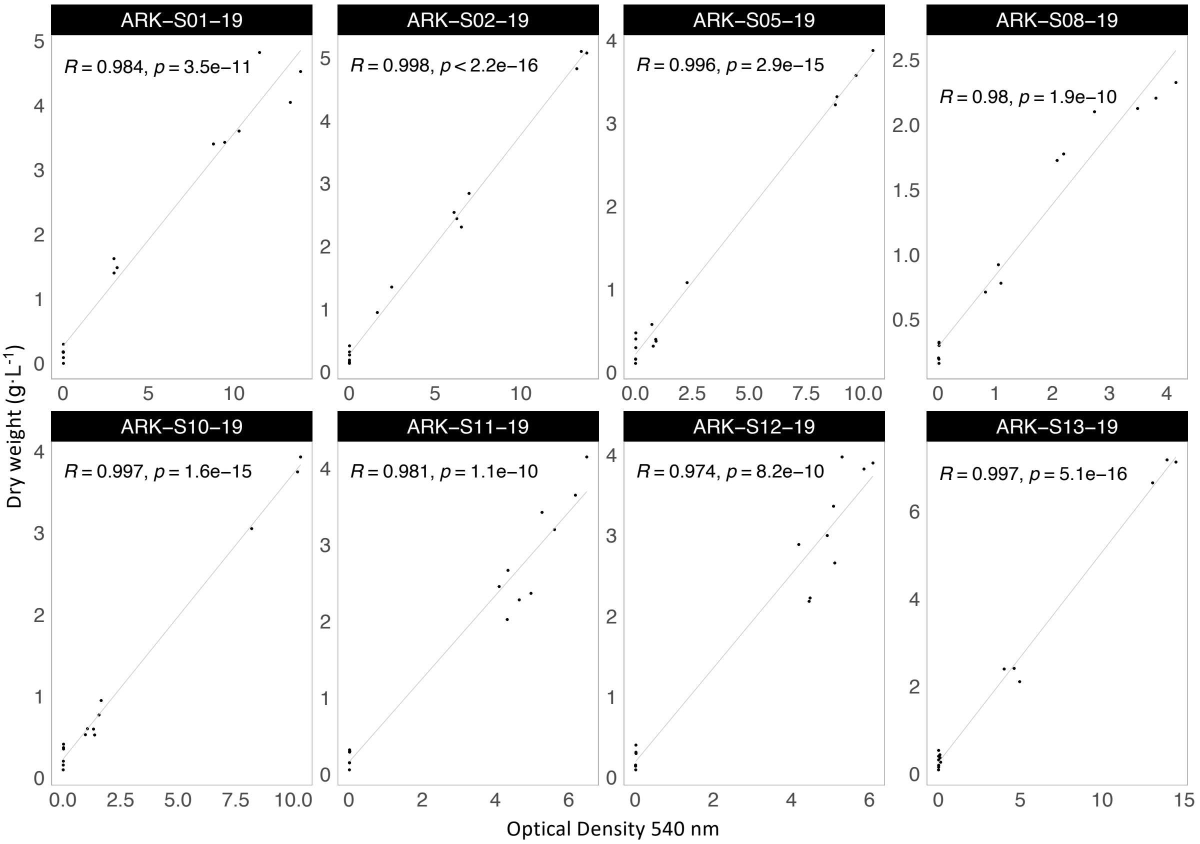

Supplementary Figure 2 (Fig. S2). The correlation between optical density at 540 nm and the dry weight ($g \cdot L^{-1}$) of eight microalgal isolates (Experiment 1). *R* is the coefficient of determination and *p* is the p-value of the regressions.

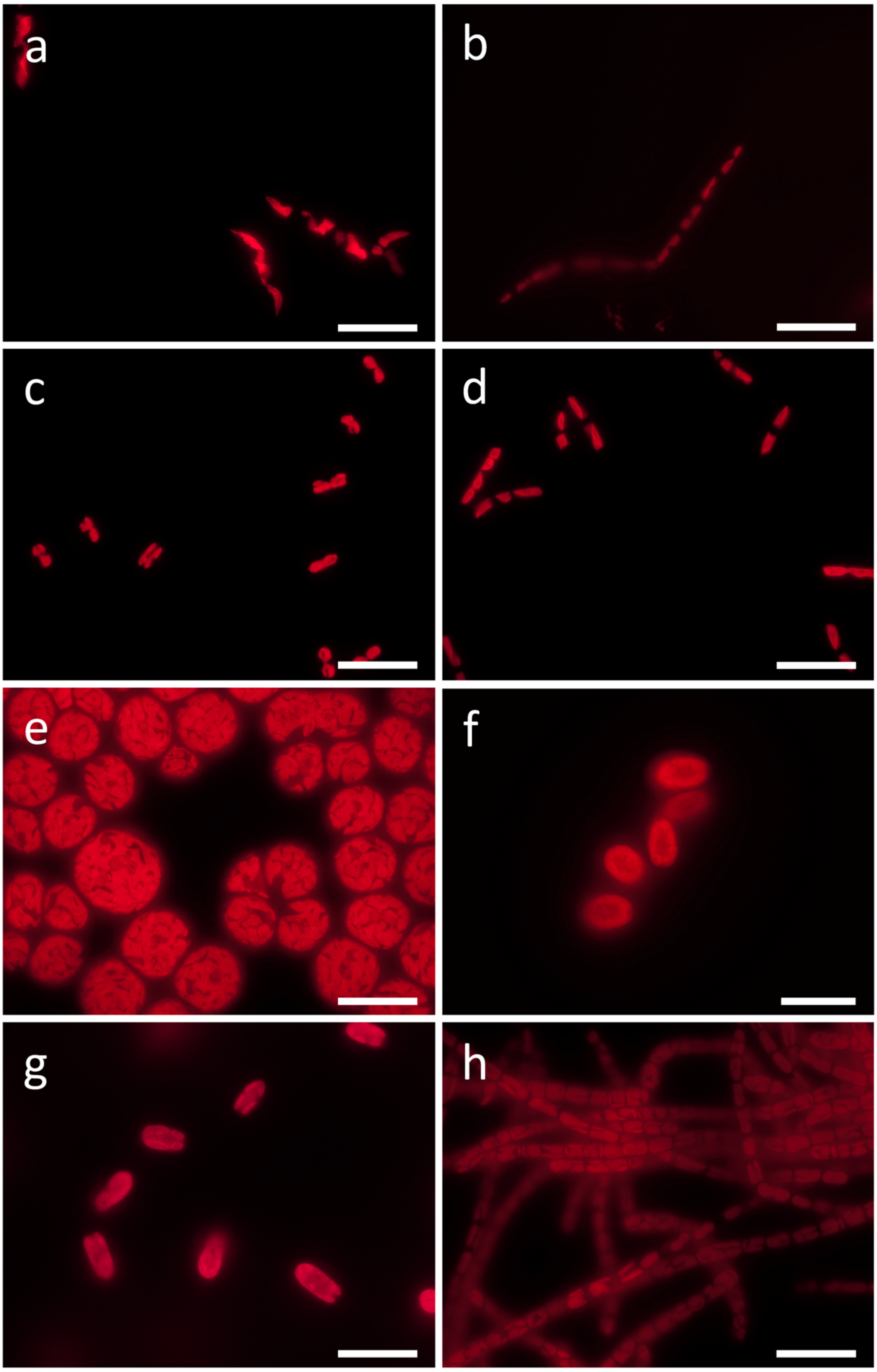

Supplementary Figure 3 (Fig. S3). Chloroplast morphology of eight algal isolates as shown by chlorophyll autofluorescence in red: *Raphidonema nivale* ARK-S01-19 (a); *Raphidonema nivale* ARK-S02-19 (b); Stichococcaceae sp. ARK-S05-19 (c); *Deuterostichococcus epilithicus* ARK-S10-19 (d); *Chloromonas* sp. ARK-S08-19 (e); *Chloromonas reticulata* ARK-S11-19 (f); *Chloromonas reticulata* ARK-S12-19 (g); *Xanthonema bristolianum* ARK-S13-19 (h). Scale bars are shown in 20 µm.

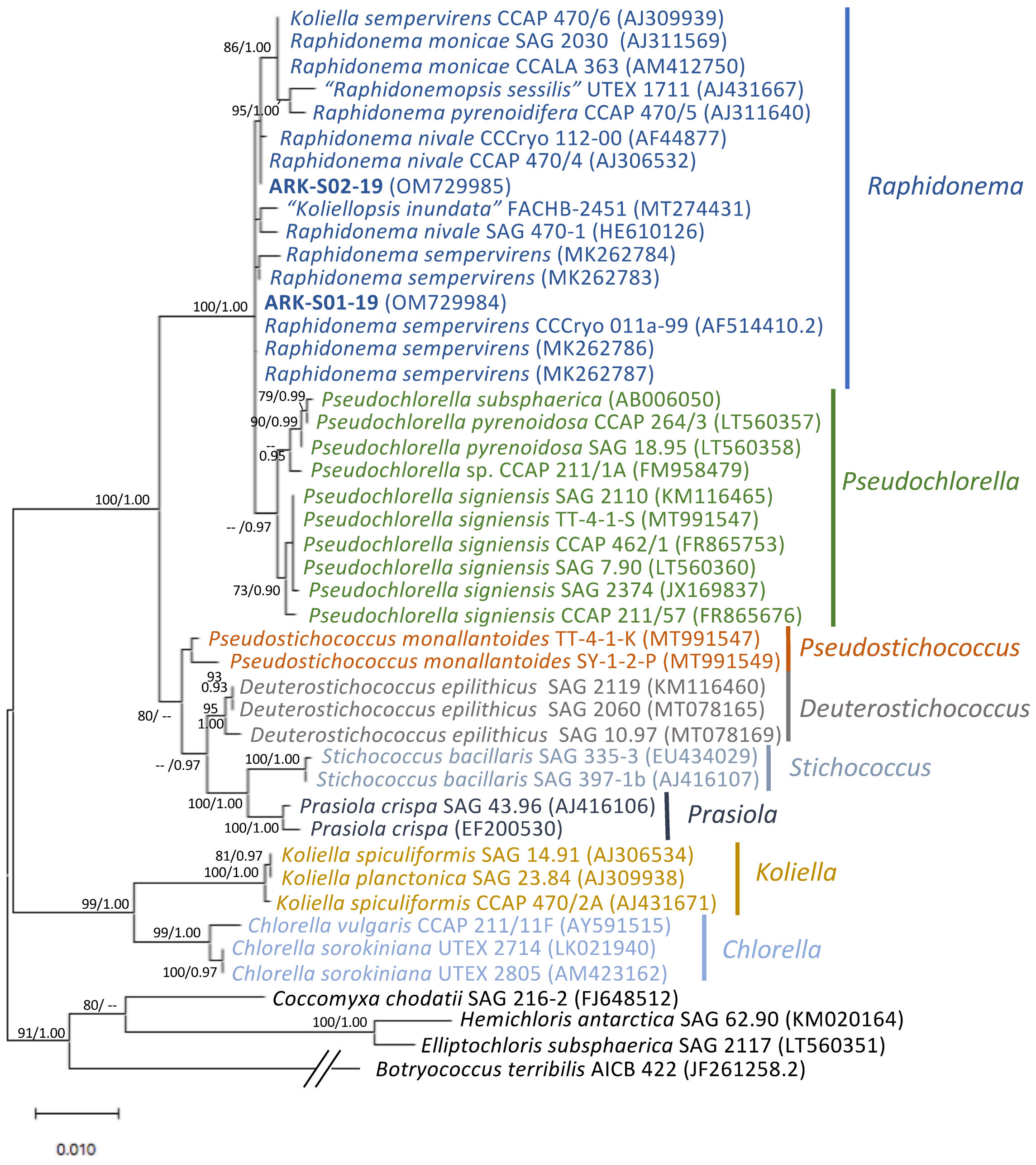

Supplementary Figure 4 (Fig. S4). A phylogenetic tree of *Raphidonema* using maximum likelihood on the alignment of 18S rRNA gene sequences. Our new isolates (sequence) are shown in bold. The best model was K2+G+I calculated by MEGAX 10.1.8. Numbers next to branches indicate statistical support values [maximum likelihood bootstraps (1,000 replicates)/Bayesian posterior probabilities]. The bootstrap support values above 70% and Bayesian posterior probabilities above 0.9 are shown. *Coccomyxa chodatii* SAG 216-2 (FJ648512), *Hemichloris antarctica* SAG 62.90 (KM020164), *Elliptochloris subsphaerica* SAG 2117 (LT560351), and *Botrycoccus terribilis* AICB 422 (JF261258.2) serve as the outgroup.

(a)

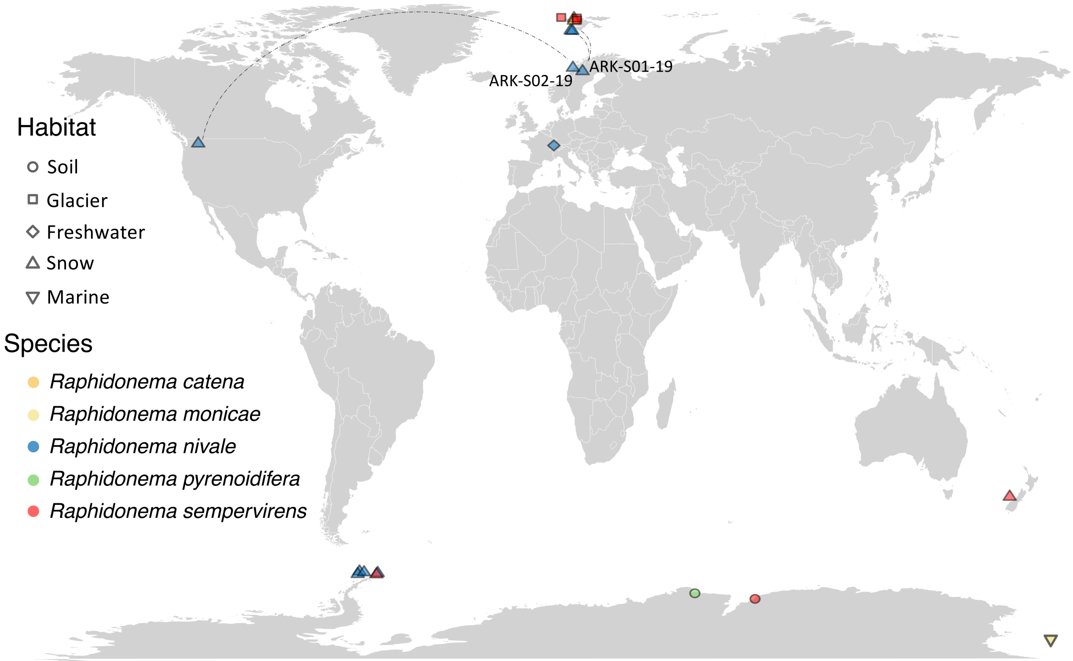

(b)

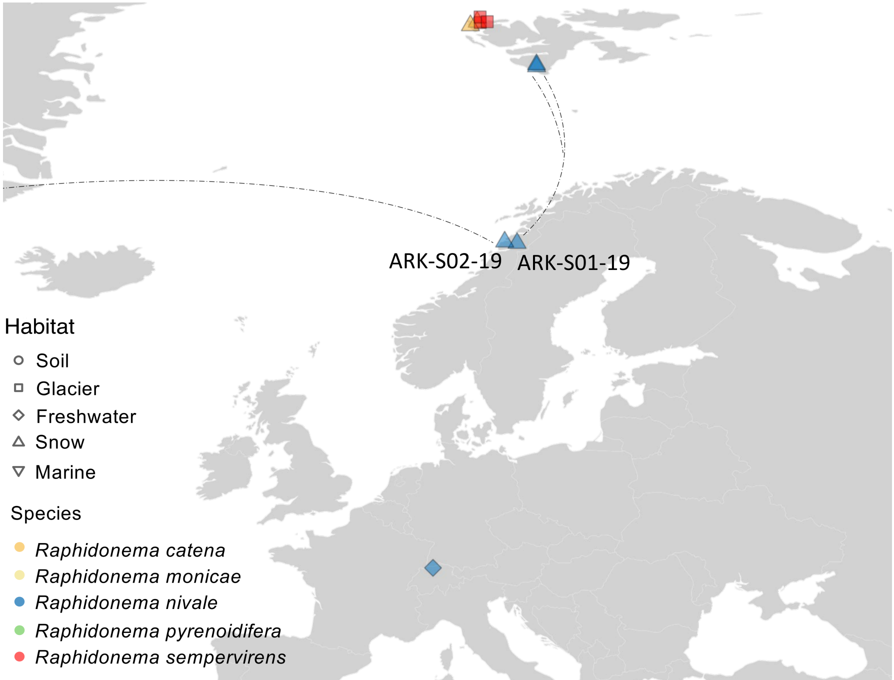

Supplementary Figure 5 (Fig. S5). The geographic distribution and habitat of each strain in the ITS2 rRNA sequence-structure maximum likelihood tree of *Raphidonema* (Fig. 3). The dotted lines indicate the closely related strains. The ITS2 rDNA sequence of ARK-S01-19 (CCCryo 558-22, OM729984) was identical to *R. nivale* strains isolated from Svalbard (CCCryo 381-11, MW077559 & CCCryo 375-11, MW077561) whilst the ITS2 rDNA sequences of ARK-S02-19 (CCCryo 559-22, OM729985) differed from that of the *Raphidonema nivale* strain (AJ431676) from Washington State (USA) by only 1 bp: (a) a world map; (b) a map mainly showing Europe.

(a)

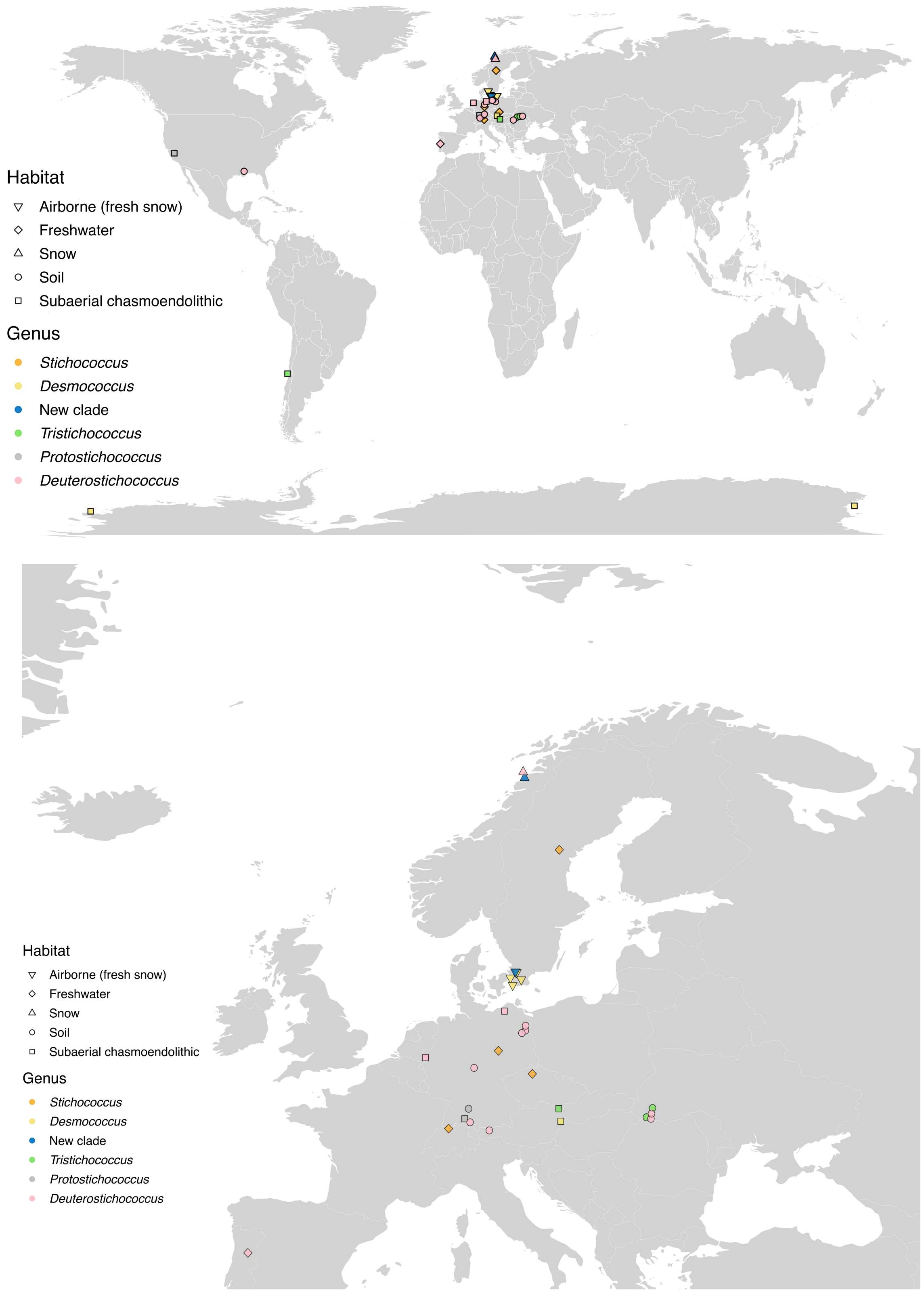
Habitat
Airborne (fresh snow)
Freshwater
Snow
Soil
Subaerial chasmoendolithic
Genus
Stichococcus
Desmococcus
New clade
Tristichococcus
Protostichococcus
Deuterostichococcus
Habitat
Airborne (fresh snow)
Freshwater
Snow
Soil
Subaerial chasmoendolithic
Genus
Stichococcus
Desmococcus
New clade
Tristichococcus
Protostichococcus
Deuterostichococcus

(b)

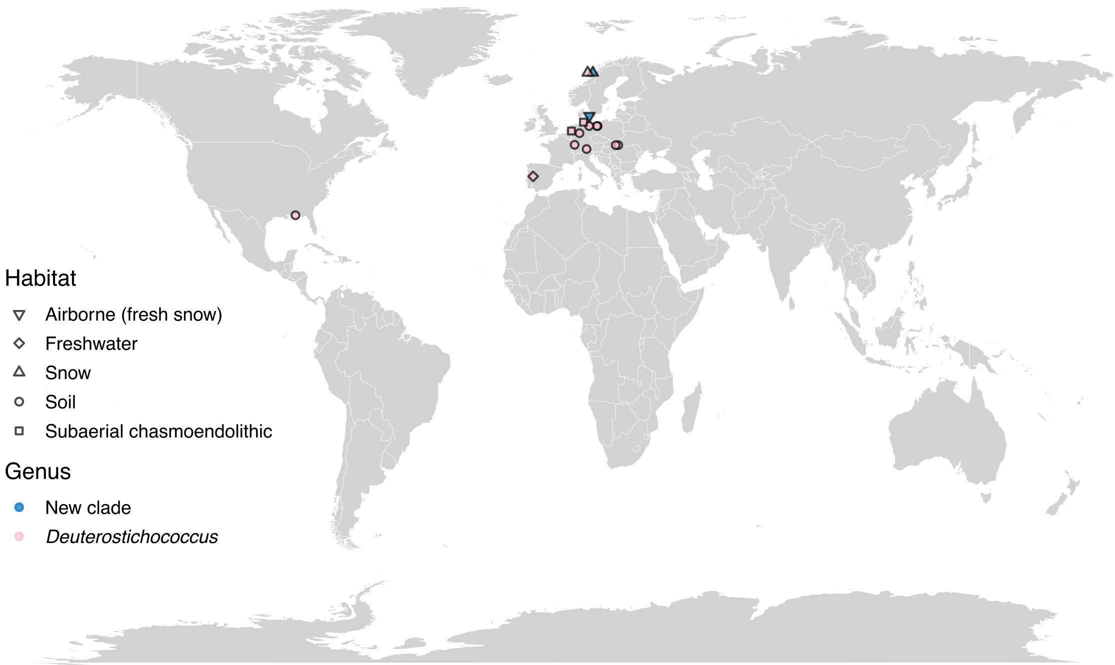

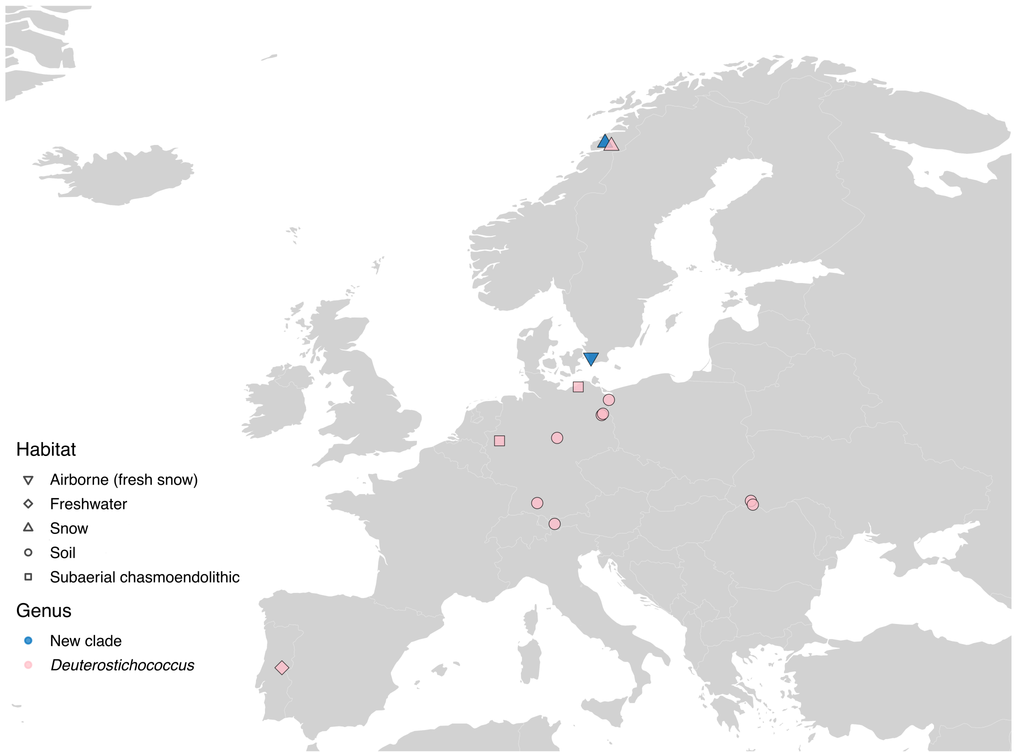

Supplementary Figure 6 (Fig. S6). A biogeographical distribution and habitat of each strain in the ITS2 rRNA sequence-structure maximum likelihood tree of *Stichococcus*-like strains (Fig. 4). (a) A world map, and a map showing mainly Europe, including all species from Fig. 4. (b) a world map and a map showing Europe that focus solely on the new clade that includes Stichococcaceae sp. ARK-S05-19 (CCCryo 560-22, OM729986), and the genus *Deuterostichococcus*.

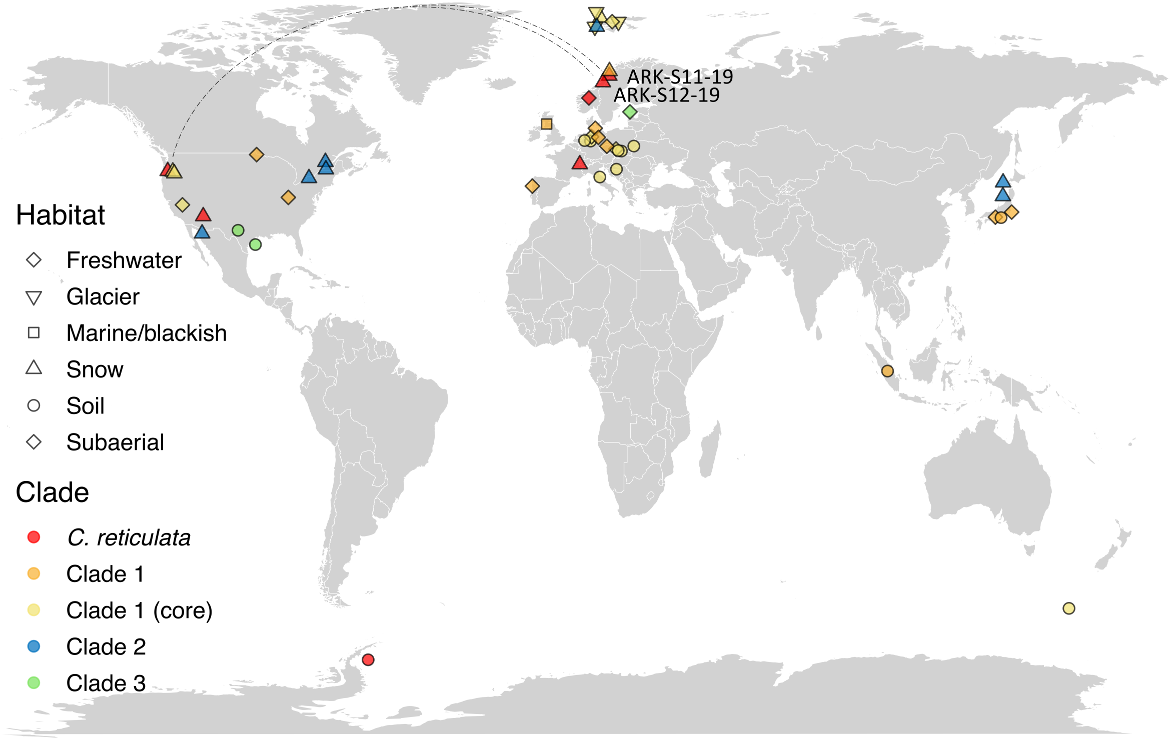

Supplementary Figure 7 (Fig. S7). Geographic distribution and habitat of each strain present in the 18S rRNA sequence based maximum likelihood tree and the ITS rRNA sequence based maximum likelihood tree of *Chloromonas* (Fig. 6). Clade 1 (core) represent the core *Chloromonas* (Barcytė *et al.*, 2018a), whilst clade 1 includes strains that belong to clade 1 excluding those in the core *Chloromonas*. *C. reticulata* including ARK-S11-19 (OM729989) and ARK-S12-19 (CCCryo 563-22, OM729990) that also belong to core *Chloromonas* are represented in red. The dotted lines indicate the strains identical to each other in both 18S rDNA and ITS2 rDNA sequences.

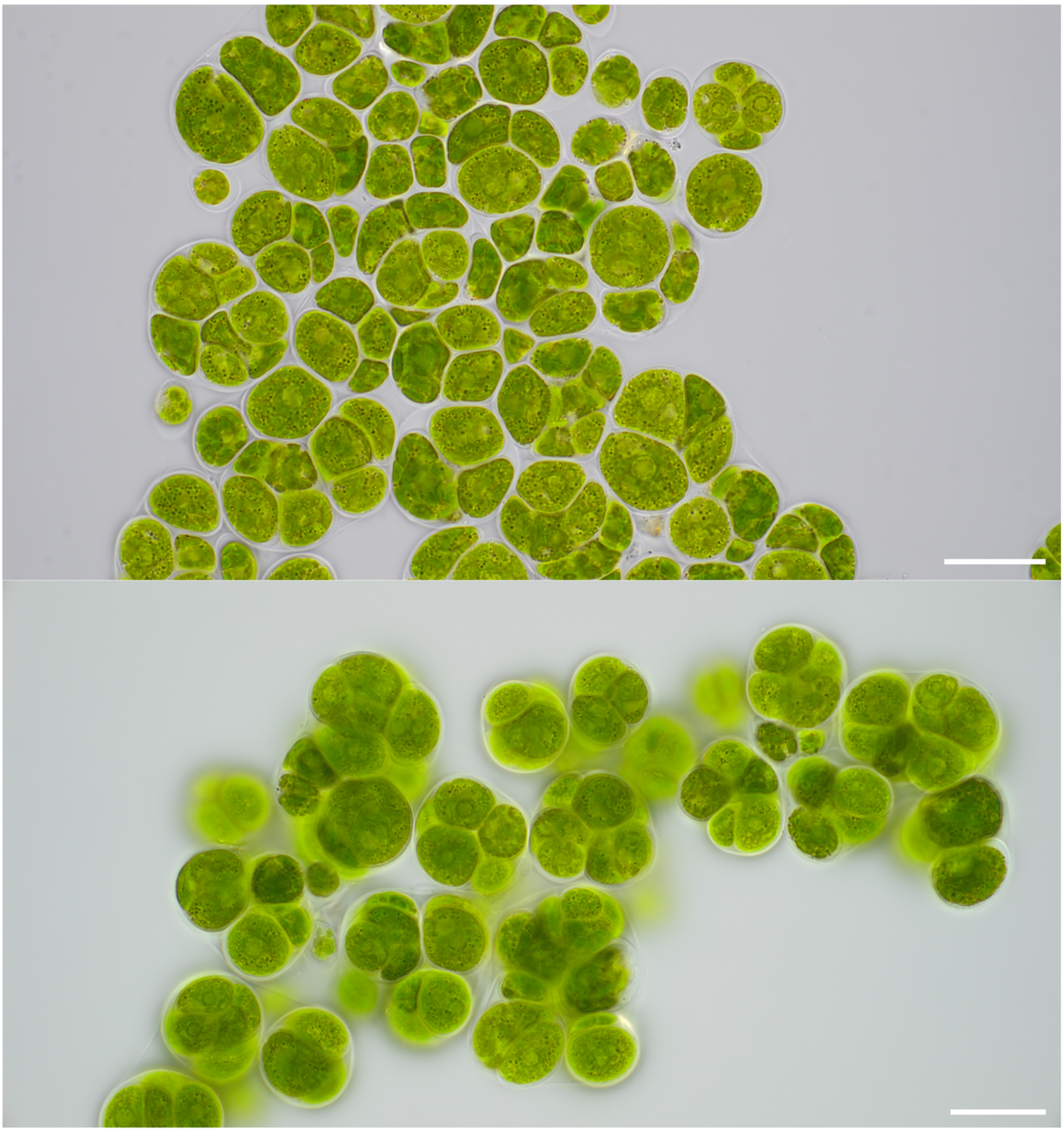

Supplementary Figure S8 (Fig. S8). Microphotographs of non-motile cells of *Chloromonas* sp. ARK-S08-19. Scale bars shown are 20 µm.

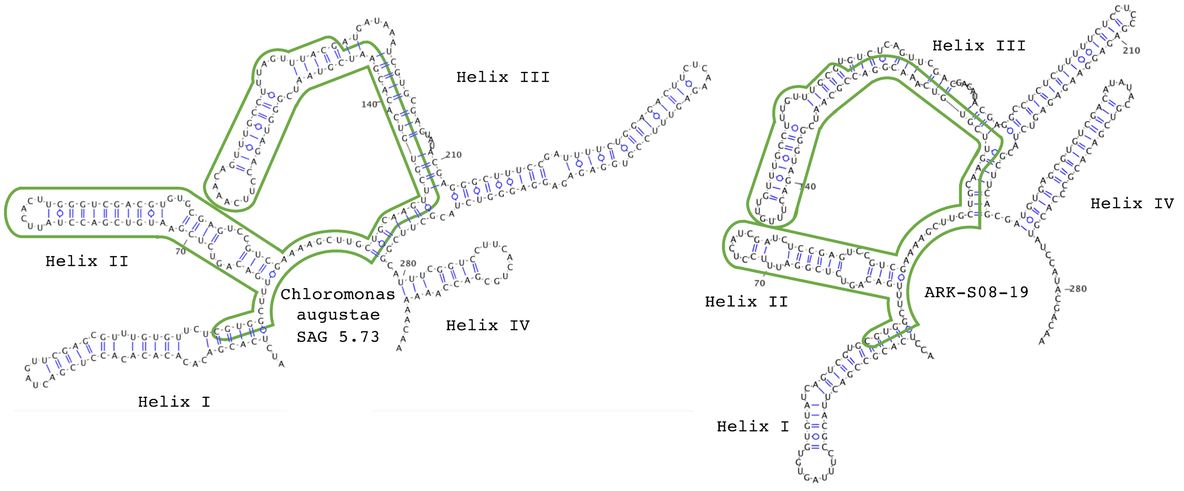

Supplementary Figure S9 (Fig. S9). The ITS2 rRNA secondary structure of *Chloromonas augustae* SAG 5.73 (AB624577, left) and ARK-S08-19 (OM729987, right). The alignable sequences between two strains were highlighted in green.

NMI-18:3 (5,9,12 – 18:3)

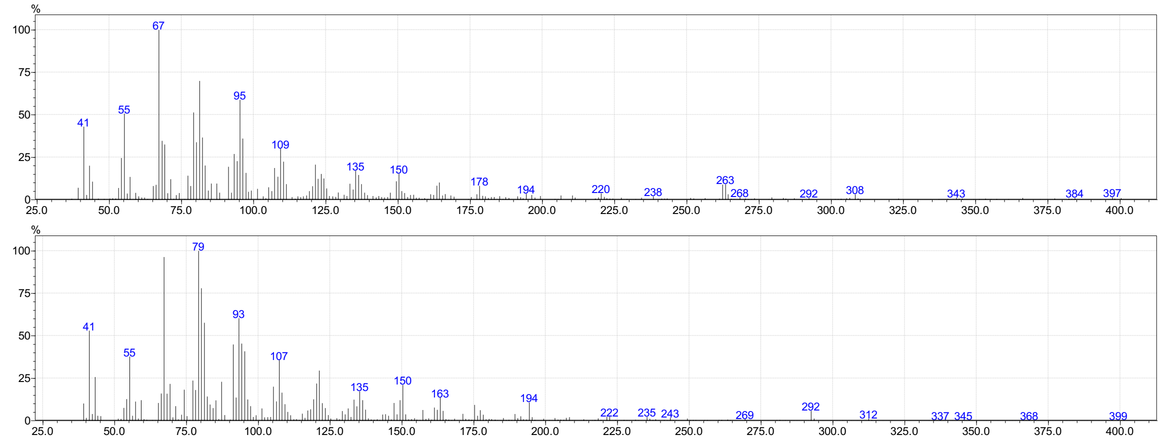

NMI-18:4

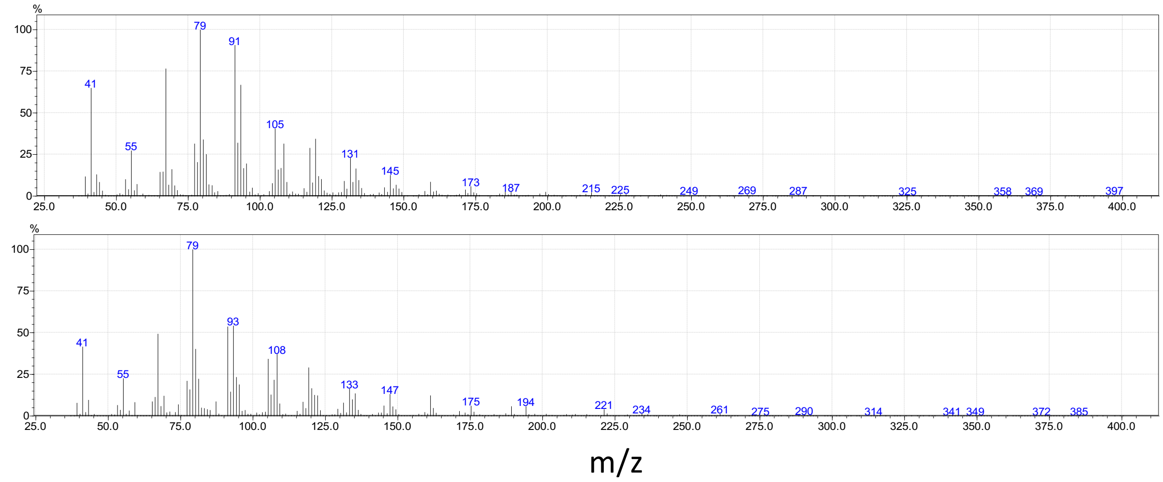

Supplementary Figure 10 (Figure S10). Mass spectrum of non-methylene interrupted (NMI) -18:3 and NMI-18:4 fatty-acids (*n=2*).

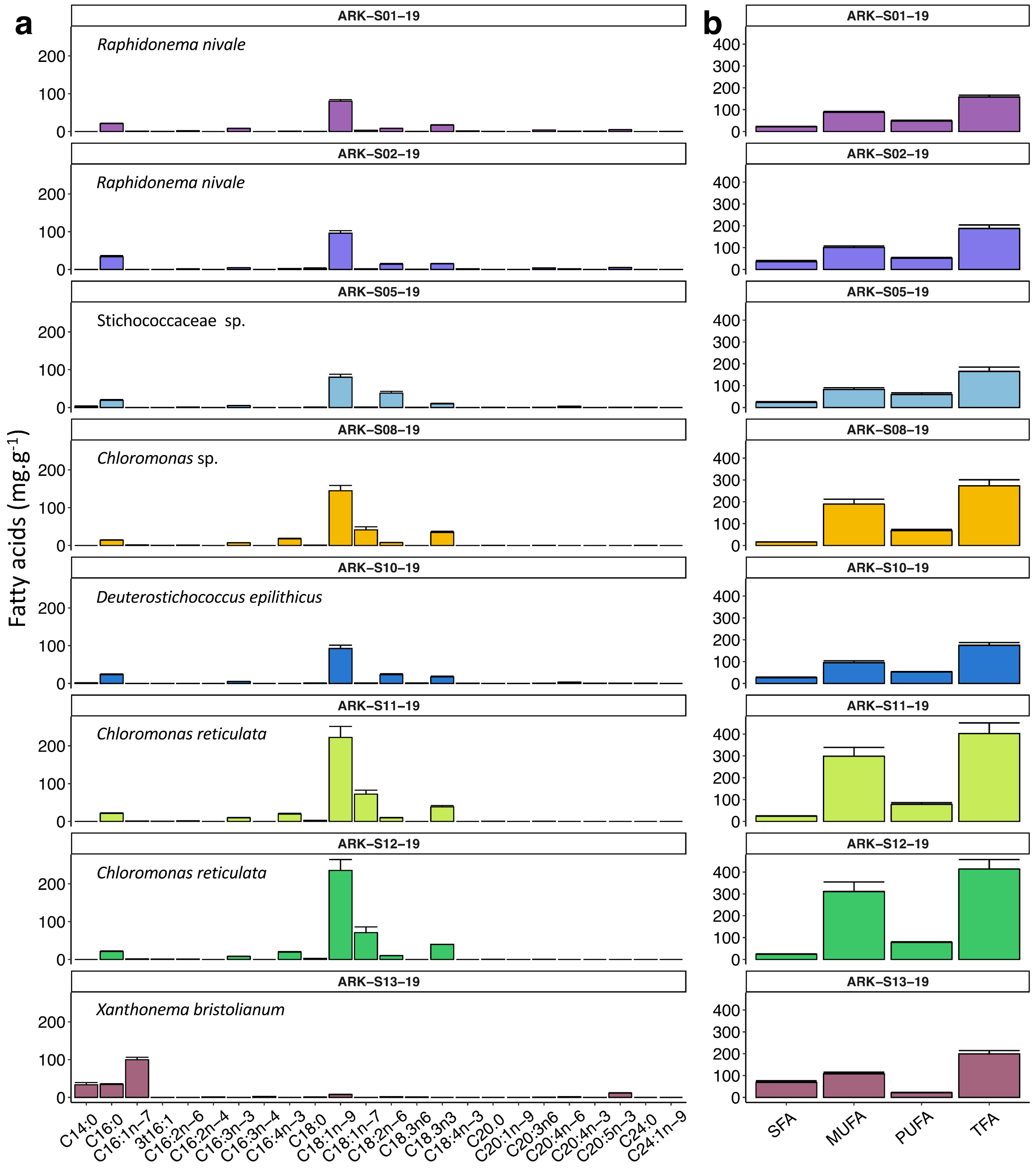

Supplementary Figure 11 (Figure S11). Fatty acid profiles of the eight isolates obtained in the stationary phase at 10°C ($mg \cdot g^{-1}$ DW) analyzed by GC-FID (Experiment 2). (a) The content of the individual fatty acids ($mg \cdot g^{-1}$). (b) The total amount of saturated fatty acids (SFA), monounsaturated fatty acids (MUFA), polyunsaturated fatty acids (PUFA) and total fatty acids (TFA). Mean values (±standard deviation) of triplicates per isolate are shown.

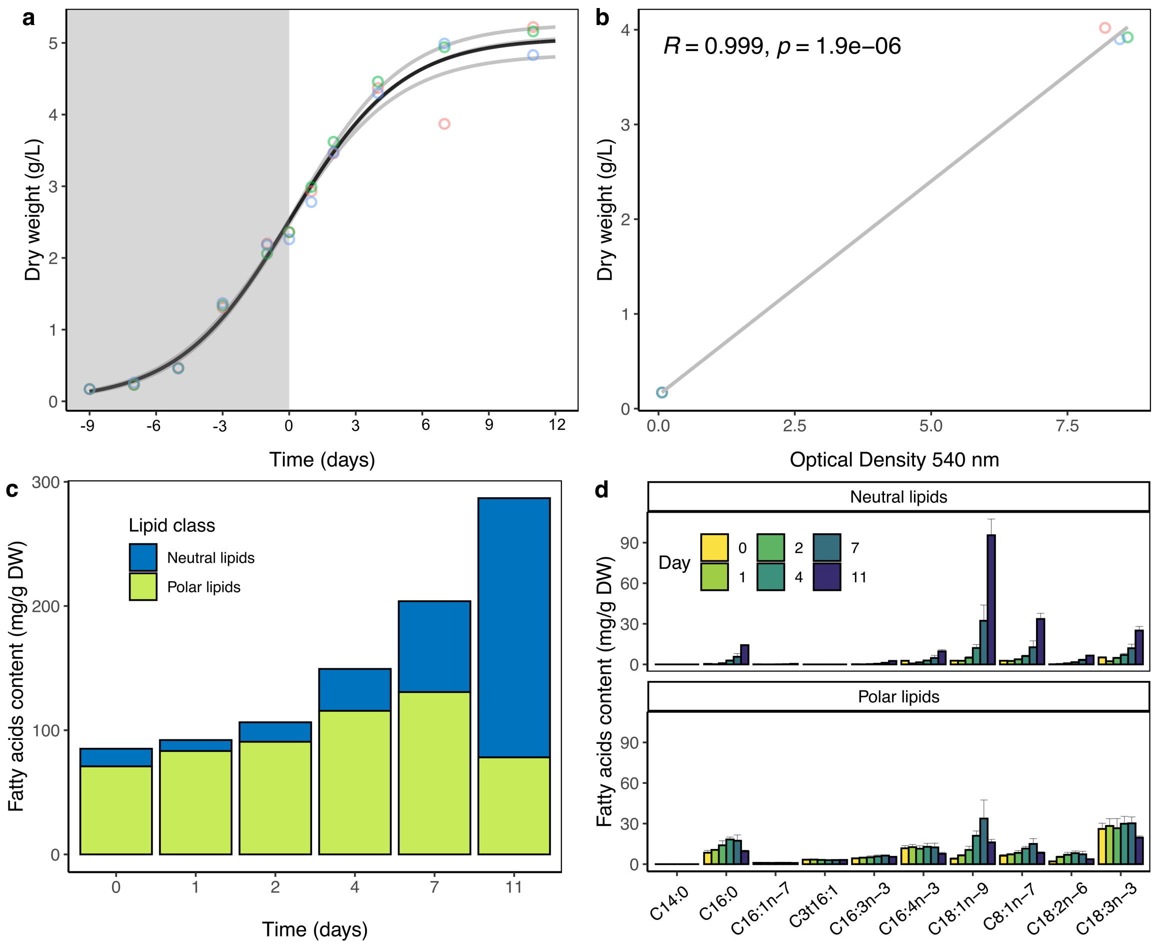

Supplementary Figure 12 (Figure S12). Growth and fatty acids content of *C. reticulata* ARK-S12-19 in Experiment 3 (n=3). Day 0 indicates the time after the cells were exposed to nitrogen starvation. (a) Cell density ($g \cdot L^{-1}$). (b) The correlation between dry weight $g \cdot L^{-1}$ and optical density at 540 nm. (c) total neutral lipids and polar lipids content from day 0 to day 11. (d) Fatty acid composition ($mg \cdot g^{-1}$ DW) from day 0 to day 11.

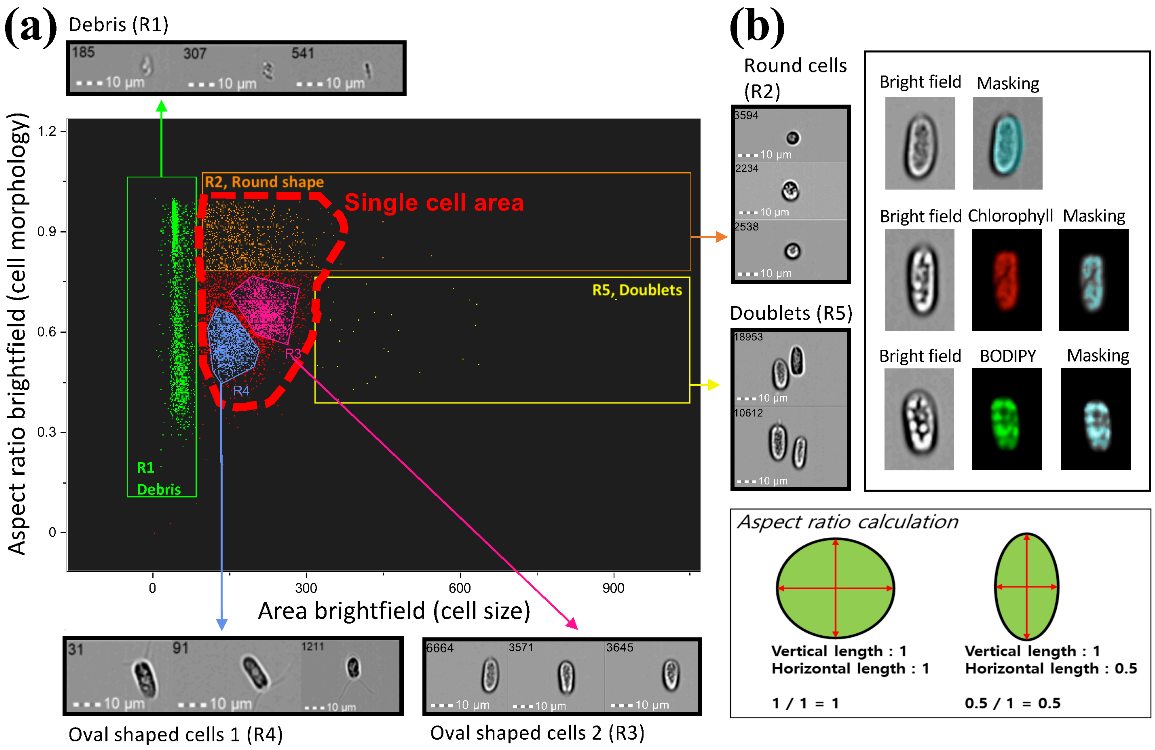

Supplementary Figure 13 (Figure S13). Imaging flow cytometer analysis: (a) Representative cell populations of *Chloromonas reticulata* ARK-S12-19 at day 0 from one biological replicate. Red marked gate was used to select the main population of single cells and exclude debris or doublets. R, region: R1, debris; R2, round cells; R3, oval shaped cells without flagella; R4, oval shaped cells with flagella. (b) Representative image of the masks that were used to measure length, width, area and fluorescence intensity of each cell. Aspect ratio is calculated based on a ratio of vertical and horizontal length of cells; round cells have the value close to 1 whilst oval shaped cells have less than 0.7. All cell images were captured with 40× objectives.

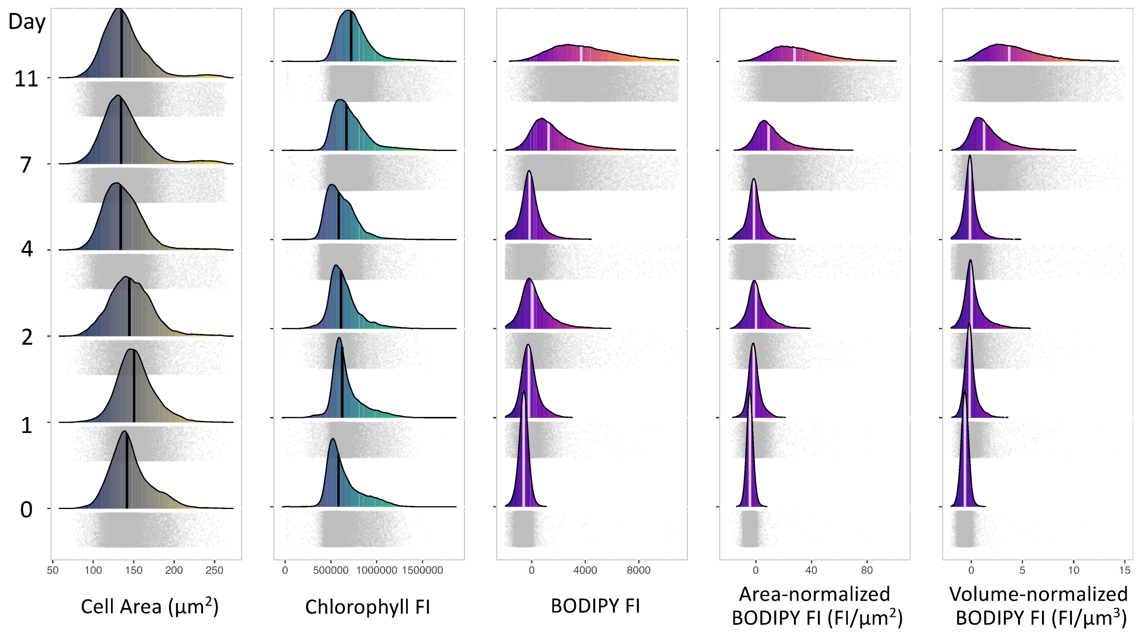

Supplementary Figure 14 (Figure S14). Cytometry data showing (left to right) the cell area (μm^2), chlorophyll fluorescence intensity (FI), Bodipy® FI, area-normalized Bodipy® FI ($FI \cdot \mu m^{-2}$) and biovolume-normalized Bodipy® FI ($FI \cdot \mu m^{-3}$) over 11 days. The vertical line within each graph indicates median of FI and grey shaded points indicate individual cell data.

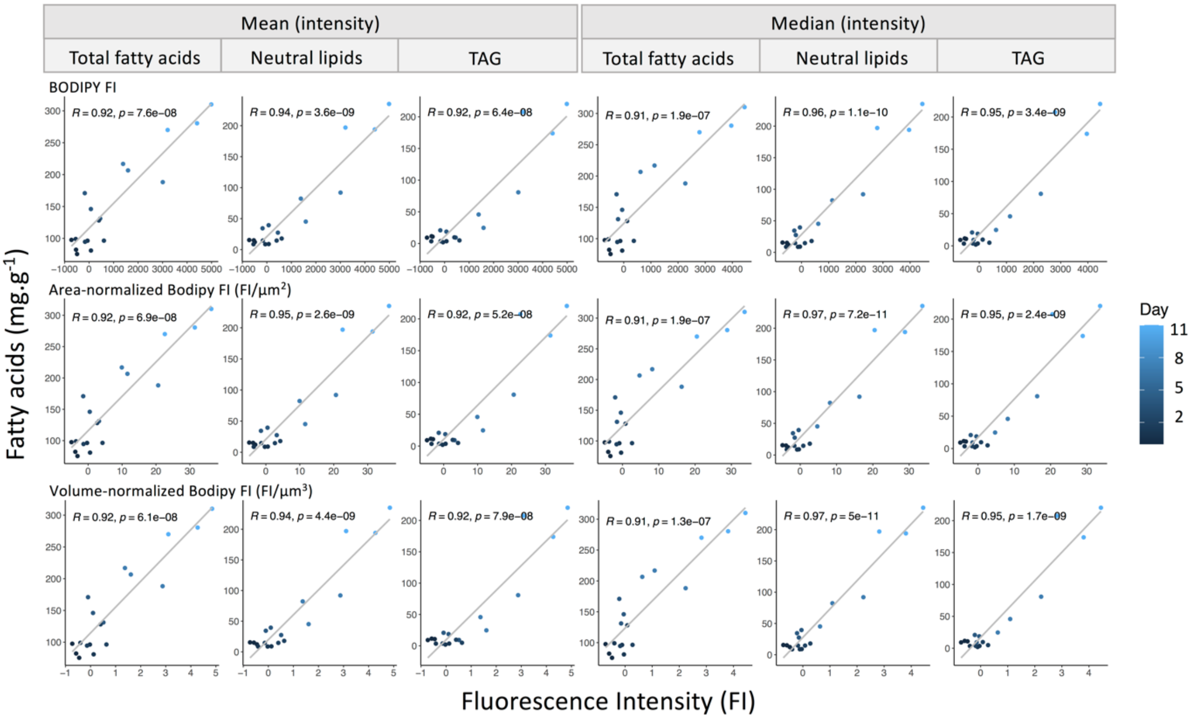

Supplementary Figure 15 (Figure S15). Correlation matrix between flow cytometry and chemical (GC-FID) analysis of lipids in *Chloromonas reticulata*. Cytometry data are either the mean or median of the normalized or non-normalized Bodipy® fluorescence intensity (FI) measured by IFC. Chemical data are either total fatty acids, total neutral lipids or TAG ($mg \cdot g^{-1}$ DW) analyzed by GC-FID.

Interpretation of Figure S15.

Both total neutral lipids (SPE) and TAG (SPE followed by TLC) were highly correlated with Bodipy® fluorescence, indicating effective and rapid quantitation of lipid droplets by IFC. The total neutral lipids had a marginally better correlation with the fluorescence compared to the TAG. This may be due to Bodipy® localizing to cell components containing diacylglycerol and monoacylglycerol in addition to TAG (Klymchenko & Kreder, 2014), or a result of the extra analytical steps introduced by TLC analysis. In either case, solid-phase extraction-based methods prove the simplest and most accurate approach. We also leveraged the image data to normalize Bodipy® fluorescence to cell area and biovolume, with slightly better results using the latter normalization approach.

Supplementary Table 1 (Table S1) A pairwise matrix of the CBCs in the ITS2 rRNA secondary structures between the groups amongst *Stichococcus, Desmococcus,* ARK-S05-19, and Prasiolales sp. S2RM26. List of strains in each group is shown in Table S2.

	1.	2.	3.	4.	5.
1. *Stichococcus bacilaris* -1	0				
2. *Stichococcus bacilaris* -2	0	0			
3. *Desmococcus olivaceus*	1	0	0		
4. ARK-S05-19	3	3	4	0	
5. Prasiolales sp. S2RM26	4	3	3	1	0

Supplementary Table 2 (Table S2). A list of strains in each group used in Table S1.

Group name	Strains	Accession number
1. *Stichococcus bacilaris* -1	*Stichococcus bacillaris* SAG 335-3	MW077554.1
	Stichococcus bacillaris SAG 379-1b	AJ431678.1
	Stichococcus bacillaris SAG 335-8	MT078154.1
	Stichococcus bacillaris SAG 379-2	HE610125.1
	Stichococcus bacillaris SAG 56.91	MT078155.1
2. *Stichococcus bacilaris* -2	*Stichococcus bacillaris* SAG 249.80	MT078156.1
3. *Desmococcus olivaceus*	*Desmococcus olivaceus* S3 F466	MK005095.1
	Desmococcus olivaceus S2RA30	MK005086.1
	Desmococcus olivaceus S2RM21	MK005089.1
	Desmococcus olivaceus SAG 25.92	KX094830.1
	Desmococcus olivaceus S2F425	MK005075.1
	Desmococcus olivaceus SAG 1.94	MT078159.1
	Desmococcus olivaceus SAG 1.92	KM020049.1
4. ARK-S05-19	ARK-S05-19	OM729986.1
5. Prasiolales sp. S2RM26	Prasiolales sp. S2RM26	MK005091.1

Supplementary Table 3 (Table S3). Additional batch growth parameters from the growth curve analysis (Experiment 1). The mid-time indicates the day at which the population density reaches 1/2*k* (which occurs at the inflection point), and generation time indicates the fastest possible doubling time. Data are mean values (±standard error) of triplicates per isolate. The asterisks indicate the mean values (± standard error) of duplicates per isolate due to an outlier that did not reach the stationary phase.

	Mid-time (day)		Generation time (d^{-1})	
	2°C	10°C	2°C	10°C
ARK-S01-19	20.7 ± 1.59	12.58 ± 0.09	1.3 ± 0.28	1.29 ± 0.03
ARK-S02-19	38.2 ± 2.73	12.07 ± 0.04	3.0 ± 0.28	1.39 ± 0.03
ARK-S05-19	35.3 ± 4.60*	15.07 ± 0.12	2.9 ± 0.24*	1.08 ± 0.004
ARK-S08-19	25.6 ± 0.82	11.42 ± 0.47	1.4 ± 0.25	1.00 ± 0.06
ARK-S10-19	43.9 ± 3.45*	14.62 ± 0.23	2.7 ± 0.21*	1.24 ± 0.05
ARK-S11-19	24.1 ± 0.26	9.24 ± 0.19	2.1 ± 0.19	0.91 ± 0.10
ARK-S12-19	24.3 ± 0.35	8.93 ± 0.25	2.0 ± 0.06	0.83 ± 0.09
ARK-S13-19	7.0 ± 0.88	13.18 ± 0.20	2.1 ± 0.09	1.55 ± 0.03

References

1. Barcytė D, Hodač L, Nedbalová L & Elster J (2018a) *Chloromonas svalbardensis* n. sp. with insights into the phylogroup *Chloromonadinia* (Chlorophyceae). *Journal of Eukaryotic Microbiology* **65**: 882-892.
2. Klymchenko AS & Kreder R (2014) Fluorescent probes for lipid rafts: from model membranes to living cells. *Chemistry & biology* **21**: 97-113.
3. Radakovits R, Jinkerson RE, Fuerstenberg SI, Tae H, Settlage RE, Boore JL & Posewitz MC (2012) Draft genome sequence and genetic transformation of the oleaginous alga *Nannochloropsis gaditana*. *Nature Communications* **3**: 686.
